# Seasonal temperatures and hydrological conditions improve the prediction of West Nile virus infection rates in *Culex* mosquitoes and human case counts in New York and Connecticut

**DOI:** 10.1371/journal.pone.0217854

**Published:** 2019-06-03

**Authors:** Alexander C. Keyel, Oliver Elison Timm, P. Bryon Backenson, Catharine Prussing, Sarah Quinones, Kathleen A. McDonough, Mathias Vuille, Jan E. Conn, Philip M. Armstrong, Theodore G. Andreadis, Laura D. Kramer

**Affiliations:** 1 Division of Infectious Disease, Wadsworth Center, New York State Department of Health, Albany, NY, United States of America; 2 Department of Atmospheric and Environmental Sciences, University at Albany, SUNY, Albany, NY, United States of America; 3 Bureau of Communicable Disease Control, New York State Department of Health, Albany, NY, United States of America; 4 Department of Biomedical Sciences, University at Albany, SUNY, Albany, NY, United States of America; 5 Center for Vector Biology & Zoonotic Diseases, Department of Environmental Sciences, The Connecticut Agricultural Experimental Station, New Haven, CT, United States of America; Columbia University, UNITED STATES

## Abstract

West Nile virus (WNV; *Flaviviridae*: *Flavivirus*) is a widely distributed arthropod-borne virus that has negatively affected human health and animal populations. WNV infection rates of mosquitoes and human cases have been shown to be correlated with climate. However, previous studies have been conducted at a variety of spatial and temporal scales, and the scale-dependence of these relationships has been understudied. We tested the hypothesis that climate variables are important to understand these relationships at all spatial scales. We analyzed the influence of climate on WNV infection rate of mosquitoes and number of human cases in New York and Connecticut using Random Forests, a machine learning technique. During model development, 66 climate-related variables based on temperature, precipitation and soil moisture were tested for predictive skill. We also included 20–21 non-climatic variables to account for known environmental effects (e.g., land cover and human population), surveillance related information (e.g., relative mosquito abundance), and to assess the potential explanatory power of other relevant factors (e.g., presence of wastewater treatment plants). Random forest models were used to identify the most important climate variables for explaining spatial-temporal variation in mosquito infection rates (abbreviated as *MLE*). The results of the cross-validation support our hypothesis that climate variables improve the predictive skill for *MLE* at county- and trap-scales and for human cases at the county-scale. Of the climate-related variables selected, mean minimum temperature from July–September was selected in all analyses, and soil moisture was selected for the mosquito county-scale analysis. Models demonstrated predictive skill, but still over- and under-estimated WNV *MLE* and numbers of human cases. Models at fine spatial scales had lower absolute errors but had greater errors relative to the mean infection rates.

## Introduction

West Nile virus (WNV) has caused 46,086 diagnosed cases in the United States, with over 2000 human deaths (1999–2016) [1]. The ecological impacts of WNV have been even more substantial, as WNV has been found in 332 bird species in the United States [2], caused a 45% decline in American Crows, *Corvus brachyrhynchos*, in the United States [3], killed millions of songbirds (e.g., an estimated 29 million Red-eyed Vireos, *Vireo olivaceus*) [4], and contributed to the listing of the Yellow-billed Magpie, *Pica nuttalli*, as ‘Near Threatened’ [5]. In addition to avian hosts, WNV has been reported from reptiles [6], mammals [7–9], and amphibians [10]. Non-avian impacts have led to substantial economic losses, notably due to infections of horses and farmed alligators [6,11].

In the Northeast, WNV is primarily found in *Culex* mosquitoes, especially *Cx*. *pipiens* (e.g., [12]). Avian hosts are thought to be responsible for the majority of WNV amplification [13,14], although species vary widely from non-infectious (e.g., Rock Pigeons, *Columba livia* [14]) to superspreaders (e.g., American Robins, *Turdus migratorius* [15]). Climatic conditions may facilitate WNV through 1) increased mosquito abundances (e.g., [16], 2) increased viral replication rates [17–19], and 3) changing the interactions between mosquitoes and their hosts. Some of these changes could be indirect, such as by affecting timing of breeding or migration for key amplifying species [20,21].

Prior studies have supported this link between WNV and climatic variables [17,22,23]. Prior studies found higher WNV infection rates with increasing temperature [22–27], including winter temperature [23], and higher infection rates with increased growing degree days [28]. Precipitation relationships have been more complex and not consistently detected [27]. Increased precipitation in the preceding year [29] and decreased current year precipitation [24] have been associated with increased WNV transmission. Precipitation may interact with temperature, as drought has been found to be important in WNV dynamics [30,31]. Warm and dry conditions during early spring have been associated with increased WNV activity [22], but not conclusively, as a range of other climate indicators, such as anomalously wet conditions in March, were also identified as potentially important in the same study [22].

Direct comparisons among studies are complicated, as studies have differed in their selection of climate variables, computation of the climate variables, inclusion of important covariates, spatial extent, and spatial resolution (see Table 1). Further, relationships between climate and disease may vary depending on geographic location (e.g., [31]). This has complicated the use of climatic information to produce robust spatial and temporal predictions of WNV prevalence. Differences in analysis extent and resolution (i.e. the scale of the analysis) have been shown to affect analysis results (the modifiable area unit problem, MAUP) [32–34]. One scale-dependent result identified from species distribution models has been that specific relationships with climate at broad spatial scales using aggregated data exist; in contrast, at finer scales other variables may dominate [35]. Indeed, this result has been observed for WNV relative to 30-year climate averages [36]. However, as the life-cycles of mosquitoes and WNV are highly temperature dependent [18,37], strong relationships with variables such as temperature and precipitation may be present even at fine spatial scales.

**Table 1:** A summary of literature that includes Connecticut or New York as part of the study area. Studies varied in their choice of dependent variable (De). Independent variables were classified as Surveillance (Su), climate (Cl), land cover (La), Sociological (So), host-related (Ho), or Other (Ot).

Study	Spatial Extent^1^	Spatial Resolution	Temporal Extent	Temporal Resolution	De^3^	Su^4^	Cl^5^	La^7^	So^8^	Ho^9^	Ot^10^
Allan et al. 2009 [38]	USA	County	2002–2004	Annual	*H*_*i*_	0	0	0	1	2	0
Andreadis et al. 2004 [39]	CT	Point	1999–2003	Annual	*H*_*c*_	1	0^6^	0	0	0	0
Andreadis et al. 2004 [39]	CT	Point	1999–2003	Annual	*M*_*a*_	0	0^6^	0	1	0	0
Bowden et al. 2011 [40]	USA	County	2002–2008	7-year period^2^	*H*_*i*_	0	0	14	0	0	0
Brown et al. 2008a [41]	New Haven, CT	Point	2004	Annual	*M*_*a*_	0	0	2	0	0	0
Brown et al. 2008b [42]	CT, DE, MA, MD, NJ, NY, PA, RI	County	1999–2006	Annual	*H*_*i*_	0	0	2	1	0	1
Brownstein et al. 2002 [43]	NY (7 counties)	Point	1999	Annual	*H*_*c*_	0	0	1	0	0	0
DeFelice et al. 2017 [44]	Suffolk, NY	County	2001–2014	Weekly	*H*_*c*_	2	0	0	0	0	0
DeFelice et al. 2017 [44]	Suffolk, NY	County	2001–2014	Weekly	*M*_*MLE*_	2	0	0	0	0	0
DeFelice et al. 2018 [45]	USA (12 counties)	County	2001–2016	Weekly	*H*_*c*_	2	6	0	0	0	0
DeFelice et al. 2018 [45]	USA (12 counties)	County	2001–2016	Weekly	*M*_*MLE*_	2	6	0	0	0	0
Diuk-Wasser et al. 2006 [46]	Fairfield, CT	Point	2001–2003	3-year-period	*M*_*a*_	0	0	97	1	0	0
Gates and Boston 2009 [47]	USA	County	2004–2006	3-year period	*H*_*c*_	0	0	1	1	0	0
Gates and Boston 2009 [47]	USA	County	2004–2006	3-year period	*E*_*c*_	0	0	1	0	0	1
Hahn et al. 2015 [24]	USA	County	2004–2012	Annual	*H*_*cz*_	0	10	0	0	0	0
Keyel et al. (this study)	NY, CT	County, Point	2000–2015	Annual	*M*_*MLE*_	5	66	4	2	7	2
Keyel et al. (this study)	NY, CT	County, Point	2000–2015	Annual	*H*_*c*_	6	66	4	2	7	2
Landesman et al. 2007 [29]	USA	County	2002–2004	Annual; Monthly	*H*_*c*_	0	6	0	0	0	0
Little et al. 2016 [22]	Suffolk, NY	13 × 13 km cells	2001–2015	Monthly	*M*_*MLE*_	0	48	0	0	0	0
Liu et al. 2009 [26]	CT	Township	2000–2005	Daily	*H*_*c*_	5	3	6	1	0	0
Manore et al. 2014 [23]	USA	County	2005–2011	Annual	*H*_*c*_	2	96	1	6	27	0
Myer et al. 2017 [48]	Suffolk, NY	Point	2008–2014	Weekly	*M*_*pa*_	0	6	37	1	0	0
Myer and Johnston 2019 [49]	Nassau, NY	Point	2001–2015	Weekly	*M*_*pa*_	1	6	16	4	0	2
Paull et al. 2017 [31]	USA	State	1999–2009	Annual	*H*_*ni*_	1	4	0	0	0	0
Rochlin et al. 2008 [50]	Suffolk, NY	Point	2000–2004	Annual	*M*_*a*_	0	0	10	1	0	1
Rochlin et al. 2008 [50]	Suffolk, NY	Point	2000–2004	Annual	*M*_*p*_	0	0	10	1	0	1
Rochlin et al. 2009 [51]	Suffolk, NY	Point	1999–2006	Annual	*M*_*a*_	0	0	3	0	0	2
Rochlin et al. 2011 [52]	Suffolk, NY	Point	2001–2004	4-year period	*H*_*c*_	8	0	30	13	0	5
Shaman et al. 2011 [25]	Suffolk, NY	13 × 13 km cells	2001–2009	Annual	*M*_*%*_	0	60	0	0	0	0
Tonjes 2008 [53]	Suffolk, NY	Zip Codes	2000–2004	Annual	*H*_*c*_	2	0	0	1	0	0
Trawinski and MacKay 2008 [28]	Erie, NY	Point	2001–2005	Weekly	*M*_*a*_	0	33	0	0	0	0
Trawinski and MacKay 2010 [54]	Amherst, Erie, NY	Point	Not reported	2–5 weeks	*M*_*a*_	0	12	66	27	0	51
Walsh 2012 [27]	NY	County	2000–2010	Annual	*H*_*pa*_	1	2	1	0	0	0
Young et al. 2013 [55]	USA	County	2003–2008	6-year period	*H*_*i*_	0	30	17	0	0	3

^1^ USA: United States of America; CT: Connecticut; DE: Delaware; MA: Massachusetts; MD: Maryland; NJ: New Jersey; NY: New York State; PA: Pennsylvania; RI: Rhode Island.

^2^ Assumed. Whether years were pooled or analyzed individually was not clear from the methods section.

^3^ *De*: Dependent variables: *E*_*c*_ equine cases; *H*_*c*_ Human cases; *H*_*cz*_ z-score deviation from mean number of human cases; *H*_*i*_ Human per-capita incidence; H_ni_ Human West Nile neuroinvasive disease cases only; *H*_*pa*_ human cases present or absent; *M*_*%*_ percent of mosquito pools testing positive; *M*_*a*_ Mosquito abundance; *M*_*MLE*_ Mosquito infection rate; *M*_*p*_ The proportion of mosquitoes belonging to a particular species, *M*_*pa*_ Presence/absence of WNV in mosquito pools.

^4^ *Su*: Surveillance variables such as number of dead birds, WNV positive birds, Human WN in previous years, Human infection rate, human immunity (estimated), mosquito infection rates from previous timepoints, mosquito abundance, absence of mosquito surveillance, site classification based on previous WNV infection rates (high, medium, low), number of complaints about mosquitoes, number of known larval sites, WNV positive mosquito pools, distance to nearest complaint, distance to nearest known larval site, distance to nearest WNV positive bird, distance to nearest WNV positive mosquito pool.

^5^ *Cl*: Climate and hydrological variables such as temperature, precipitation, growing degree days, and anomalies for each of these variables. Often calculated as minimum, mean, maximum, or cumulative values for different time periods (e.g., month, season, year).

^6^ Temperature and rainfall values were discussed, but not statistically related to the WNV results.

^7^ *La*: Land cover variables such as percent/proportion land cover for different land cover types, buffer distances, or administrative units or distance to land cover features. Soil drainage characteristics were also included here, as were Normalized Vegetation Difference Index (NDVI), Disease Water Stress Index (DWSI), and Middle Infrared Band.

^8^ *So*: Sociological variables such as age (median), education, employment (percent), household income (median), housing age, human population (density), human population (total), race, senior households (count, >65), septic systems (count), vacant housing (percent), urban or rural (categorical).

^9^ *Ho*: Host variables such as avian abundance (e.g., by order or species), avian diversity, and community competence.

^10^ *Ot*: Other variables, such as aspect, catch basin area, catch basin count, county area, elevation, equine density, flood zone, flood zone (distance to nearest), road length, road polygons (index of fragmentation), slope, wastewater treatment plants (distance from, count per administrative unit), year.

In this study the main goals were to explore the spatial and temporal relationships between climate and WNV and develop a well-validated statistical model that included climatic as well as non-climatic environmental factors for the northeastern United States. We hypothesized that climatic variables would be important at both coarse (county) and fine (point) spatial scales. Conversely, WNV is widely distributed across many different climatic regions [24,56], and therefore we tested the alternative hypothesis that WNV prevalence and human cases do not depend on climatic variables, and that previous results were due to an omitted, correlated covariate.

## Methods & data

### Overview

We fit models at two spatial scales, with and without climate variables, and examined the error for a new year of data. We examined statistical relationships between two dependent variables, WNV mosquito infection rates (*MLE)* and human cases of WNV (see *Dependent variables* section), and 86–87 independent variables, grouped into *climate* (66 covariates), *surveillance* (5–6 covariates), host (7 covariates), human population (2 covariates), land cover (4 covariates), and wastewater treatment (2 covariates). Human population, land cover, and wastewater covariates were snapshots in time and were treated as constant across time. Dependent variables were related to independent variables using a random forest analysis [57]. The analysis was conducted with data aggregated over entire years. For *MLE*, relationships were evaluated at two spatial scales (county and trap, see *Scales of analysis*, below), while for human data, due to data restrictions related to privacy concerns, only the county-scale data were used. We used a leave-one-year-out cross validation approach, where the random forest model was fitted using data from all years except one, and the resulting model was used to predict the remaining year. See *Statistical Approach* below for full details. Data processing was performed using Python 2.7 [58], ArcGIS 10.6 (ESRI, Redlands, CA), and R 3.4.3 [59]. All statistical analyses were performed in R [59]. Descriptive information for non-categorical covariates are included in S1 File and a Data Dictionary describing the variables in S2 File. Text files used to run the analyses at the county scales are includes in zipped format as S3 File. For trap-scale data, contact the New York State Department of Health [60] and the Connecticut Agricultral Experimental Station [61] as the trap locations contain sensitive information.

### Scales of analysis

Data were analyzed at two scales: aggregated by county (hereafter the county-scale) and from individual mosquito trap locations (hereafter the trap-scale). Due to high uncertainty in some *MLE* for some trap locations (see *Dependent variables* below for *MLE* calculations), trap-scale data were analyzed in two ways, 1) including *MLE* with large confidence intervals, and 2) excluding *MLE* where the estimated 95% confidence interval exceeded 15 infected mosquitoes per 1000 (hereafter the trap-scale subset). Human cases were also analyzed at two different extents: all counties in both New York and Connecticut (hereafter: human all counties) and for just those counties for which mosquito surveillance data were available (hereafter: human subset).

### Dependent variables

#### Human cases

Human case data, aggregated by county and year, were obtained for the entire state of New York (NY) for 2003–2015 [62] and for the state of Connecticut (CT) from 2000–2015 [63]. Data from both states were pooled for the analysis as results were qualitatively similar when each state was analyzed separately (results not shown). Cases of West Nile Fever and West Nile Neuroinvasive Disease were pooled to increase the sample size, as these two manifestations are highly correlated [40] and this approach has been found to have greater prediction accuracy [55]. West Nile Fever corresponds to clinical cases where the symptoms include fever [64], but the cases were not neuroinvasive. West Nile Neuroinvasive Disease included cases of meningitis, encephalitis, and meningoencephalitis [64]. We note that mild cases of West Nile Fever may go unreported, as the majority of human WNV infections (~80% [65]) do not cause any detectable symptoms, and <1% are neuroinvasive [65,66]. Therefore, the reported cases represent a very small fraction of the human WNV infections. Further, case locations correspond to the patients’ county of residence and may not indicate the county where the disease was contracted.

We used total cases as our dependent variable instead of incidence as this approach makes no assumptions about the relationship between West Nile virus cases and total human population. Humans are dead-end hosts for West Nile virus, and therefore do not amplify the virus. As a consequence, the contact rate between mosquitoes and humans is far more important than total human population for determining cases, and this contact rate varies non-linearly with human population. We present a subset of our analyses using incidence in S4 File for comparison purposes.

#### Mosquito infection rates

*MLE* were calculated based on mosquito trap data. We obtained data from 8 counties in CT [61] and 8 counties in NY State (one western, two central and five southeastern counties) [60]. The data cover the years 2000–2015 (not all data available for all years). These data were pooled from several different mosquito control programs, and each agency employed a slightly different sampling design (see S5 File). For our analysis, any mosquito pool from a CDC Gravid trap [67] that was deployed for less than 24 hours and was tested for WNV was included in the analysis. We restricted the analysis to *Culex pipiens*, *Cx*. *restuans* and *Cx*. *salinarius*, as empirical data have demonstrated that *Cx*. *pipiens* and *Cx*. *restuans* can amplify WNV [68–71] and *Cx*. *pipiens* and *Cx*. *salinarius* can serve as bridge vectors to humans [12,71–73], especially in the northeast [12,39,73,74]. *Cx*. *pipiens*, *Cx*. *restuans* and *Cx*. *salinarius* were pooled in the analysis, because the NY mosquito sampling protocol does not distinguish between *Cx*. *pipiens* and *Cx*. *restuans* due to issues with species identification [75]. Mosquito identifications were based on one or more standard references [76–80]. WNV *MLE* were calculated using Maximum Likelihood Estimates [81,82] in R [59,83]. Maximum Likelihood Estimates calculate a mean infection rate and 95% confidence intervals based on the distribution of positive mosquito pools and the number of mosquitoes in each pool and represents a substantial improvement over estimates based on minimum infection rate [81]. We note that the estimates obtained using R for some samples deviated from the estimates obtained with the standard CDC Excel plugin [84], likely due to the omission of a bias correction term in the R version. However, we viewed the magnitude of these inconsistencies as relatively minor compared to the increased convenience of computing the infection rates using R. Samples were pooled for each year based on spatial location. At the trap-scale, 200 m buffers were generated surrounding all trap locations, and any overlapping buffers were treated as a single trap location. This was necessary to correct for minor inconsistencies in the reporting of trap locations across years (e.g., the same trap location may have had a new GPS point collected each year, and in some cases this point may have corresponded to the actual trap location, whereas in other cases this may have corresponded to the center of the area being sampled by the trap). A visual inspection suggested that the use of 200 m buffers (potentially merging traps 400 m apart) adequately addressed these issues, while still maintaining spatial proximity to the original locations.

### Covariates

#### Climate (66 covariates)

Climate data were derived from gridded ensemble estimates of daily temperatures and precipitation at 1/8° resolution (~12 km × 12 km) for all years included in this study (2000–2015) [85]. The data are available at http://dx.doi.org/10.5065/D6TH8JR2. The gridded data are based on the Global Historical Climatology Network-Daily dataset (GHCN-Daily [86,87]) using daily precipitation and temperature data, with supplemental data from the meteorological observations from the U.S. Natural Resources Conservation Service (NRCS) Snowpack Telemetry (SNOTEL). Daily average temperature and daily temperature range (daily maximum–daily minimum) were interpolated applying a distance-weighted station averaging model. Over the Continental US (CONUS) domain a total of 12,153 (8953) stations provided precipitation (temperature) observations [85]. Precipitation was processed using a similar distance-weighted averaging method. In this particular method the interpolation of precipitation is divided into two components (a) the probability of precipitation (PoP) and (b) the precipitation amount. Furthermore, the method applies an ensemble (therefore probabilistic) interpolation approach, which accounts for the residual variance. This improves the representation of local extremes compared with other gridded daily temperature or precipitation data products. For more details see [85,88].

For the county-scale, climate data were extracted for the county centroids (see S6 File for a justification of the use of the centroid). At the trap-scale, climate data were extracted for the centroid of the merged trap buffers (see *Dependent variables* above, typically the trap location). We aggregated the climate data into four quarters (January–March; April–June; July–September; and October–December). While the majority of WNV cases occur from July to September, we included early season data to account for processes related to the emergence and amplification of WNV, while the late season variables were included to address the end of the WNV season. For each quarter, we calculated the cumulative growing degree days (relative to 10°C), cumulative precipitation (mm), average precipitation intensity (mm day^-1^), minimum daily temperature (°C), maximum daily temperature (°C), mean minimum temperature (°C), mean maximum temperature (°C), and diurnal temperature range (°C). Minimum and maximum daily temperature correspond to the lowest/highest record observed on a day within the period, while mean minimum and maximum temperature correspond to the average minimum/maximum temperature for the entire period. Growing degree days for quarter 1 were omitted as there were not many days above 10°C during this three-month period leading to very little variation in this variable. We note that much of the fourth quarter corresponds to the time period after the mosquito season was effectively over, but it was included to capture any effects related to the ending or possible extension of the time when mosquitoes were active.

Growing degree days were calculated as the cumulative number of degree days above 10°C within the 3-month quarter. Specifically, for each day, 10°C was subtracted from the mean temperature. If this reduced the value to below zero, zero was used instead, and the sum of all these values for each quarter was computed. We chose 10°C, as this temperature limit was used in one prior study in the region [26], although a similar rationale could have been used to select 15°C [28,74]. However, we expected that data from either of these two thresholds would be very highly correlated, so we did not explore the 15°C threshold. Precipitation intensity was calculated as the total precipitation for each quarter divided by the number of days it rained in that quarter [89]. A minimum of 0.254 mm of rainfall, the threshold of common instrument measurements, was required to count as a rain day.

Daily soil moisture was taken from the NLDAS Soil Moisture 0–200 cm soil depth [90–93], and aggregated to quarterly averages and quarterly means. These were used to calculate drought anomalies. The quarterly averages were extracted for county centroids and trap locations as above.

Climate anomalies were calculated for each climate variable with respect to a baseline mean for the study period (2000–2015). Although the World Meteorological Organization 30-year baseline is defined as 1981–2010, the use of the study period as a baseline leads to a mean anomaly of zero, which improves the interpretability of the results, and prior studies comparing a study mean to an alternative time period found no meaningful difference [23]. For temperature, the anomaly was calculated as deviation from the mean (Eq 1), whereas for precipitation and drought the anomaly was calculated as percent deviation of the mean (Eqs 2 and 3).

$$T_{anomaly}=T_{quarterly}-\bar{T}$$ (1)

$$P_{anomaly}=\frac{{(P}_{quarterly}-\bar{P})}{\bar{P}}\times 100$$ (2)

$$D_{anomaly}=\frac{{(D}_{quarterly}-\bar{D})}{\bar{D}}\times 100$$ (3)

*T*, *P*, and *D* correspond to temperature, precipitation, and drought respectively, and the subscript anomaly refers to the anomaly values for a given year, quarterly refers to the quarterly value for that year, and the bar indicates the quarterly mean across all years.

#### Surveillance (5–6 covariates)

We included trapped mosquito abundance to control for any effects of mosquito population size. Unfortunately, some heterogeneity in this variable exists, i.e., in CT, all mosquitoes captured were tested for WNV, whereas in NY, a maximum of 90 pools of up to 50 mosquitoes were tested (see S5 File for more details). Consequently, the NY data may underestimate the true sampled abundance at the trap. We included abundance divided by the number of pools tested (hereafter called density), to control for unequal sampling efforts across counties and years. The bait used in the CT gravid traps changed from a grass/sod infusion [94] in 2000 and 2001 to a rabbit chow infusion (Purina Mills LLC, St. Louis, MO) for 2003–2005 [39], and was switched to a hay/yeast/lactalbumin infusion [95] starting in 2006. Baits were unspecified for NY counties, with the exception of Suffolk, where a rabbit chow infusion was used [25]. Consequently, a BAIT covariate was added to the analysis as a factor with four levels: unspecified, grass/sod, rabbit chow, and hay/yeast/lactalbumin. The first level, unspecified, was used as a reference level, and was not counted towards the number of covariates. *MLE*, the dependent variable in the mosquito analyses, was included as an independent variable in the human subset analysis.

#### Host (7 covariates)

West Nile virus is an enzootic virus, and is mainly amplified by birds [70]. We obtained avian abundance information from the Breeding Bird Survey (BBS) for each state by year [96]. We included five species for which we had data, that have been identified as especially important in the transmission of WNV: American Robins (*Turdus migratorious*) [15,70], Blue Jays (*Cyanocitta cristata*) [97], Northern Cardinals (*Cardinalis cardinalis*) [70,97], House Sparrows (*Passer domesticus*) [15,70,97] and American Crows (*Corvus brachyrhynchos*) [14]. In addition, we included two aggregated covariates: the total avian abundance and the host reservoir competence index, weighted by abundance. The weighted host competence index (*Eq 4*) was calculated by taking each species’ abundance (*a*) multiplied by its host reservoir competence index value. Host reservoir competence index values are the product of susceptibility (*s*, the proportion of birds that become infected as a result of exposure), mean daily infectiousness (*i*, the proportion of exposed vectors that become infectious per day), and duration of infection (*d*, the number of days that a bird maintains an infectious viremia)[14,98]. This approach is similar to the approach taken by Kilpatrick et al. [15], but we omitted the feeding preference term due to lack of data.

$$C_{w}=a*s*i*d$$ (4)

The host reservoir competence indices were extracted from the literature [14,97]. Any species without host competence information was excluded. The species in the host competence index included on average 57% percent of the total species abundance. We note that the BBS data are limited, as these data were estimated at the state level (in contrast to Manore et al. [23], who used route-level data to estimate abundances for individual counties). The point count survey method employed by the BBS has also been critiqued on statistical grounds (e.g., [99]).

#### Human population (2 covariates)

Based on previous research, human population is associated with both human cases and *MLE* [39]. Total human population based on the 2010 US Census [100] was obtained for counties (county-scale) and the census tracts containing trap centroids. Human population was converted to population density by dividing by county or tract land area.

#### Land cover (4 covariates)

Based on the results of a prior study [101], we examined the proportion of urban, forest, open, and wetland land cover within each county (county-scale) or within 1000 m of the trap site (trap-scale) using the National Land Cover Data 2011 [102]. A 1000 m buffer was previously identified as being consistently associated with land cover variables [54], but see [46].

#### Wastewater treatment (2 covariates)

Locations of wastewater treatment facilities were obtained from the United States Environmental Protection Agency [103]. These were classified as major or minor. At the county-scale, the number of major facilities and the total number of facilities (highly collinear with the number of minor facilities) were included in the analysis. At the trap-scale, the distance to the nearest major wastewater treatment facility and the distance to any wastewater treatment facility were included in the analysis.

### Statistical approach

Correlations were calculated for each of the spatial scales (S7 File). Although correlations among covariates varied substantially (e.g., mean *r*_*p*_ = 0.08; 0.00005–0.99 min–max; mosquito infection rates, county scale), no variables were excluded on this basis.

We chose to analyze our data using random forest models [57,104], as preliminary analyses showed random forest approaches had similar or better predictive capability when compared to linear methods (GLMs). Random forest methods take a sample of the data set (with replacement) and construct a cartographic regression tree based on the sample. This approach was then repeated many times (see numbers of trees below) and the final results were obtained by averaging across all trees. Variable importance was assessed using permutation approaches that randomly changed input variables and examined the magnitude of the change in the resulting predictions [57]. For each random forest analysis, the number of variables to try at each split (m) was reached by trying each combination and using the value that corresponded to the best *R*^*2*^ value. We used 500 trees for screening for m values, and 5000 trees for the final analyses. Each tree had a single terminal node. We evaluated model fit (see *model fit statistics* below for definitions) by examining 1) the root mean squared error (RMSE), 2) RMSE scaled by the mean value, 3) the coefficient of determination, *R*^*2*^, or the percent of variation explained by the model in the validation set, [105], 4) the Spearman Rank correlation coefficient (*r*_*s*_) between the predicted and observed values, and 5) the Pearson correlation coefficient (*r*_*p*_) between the predicted and observed values.

It has been recommended to run random forest models twice: once with all variables of interest, and a second time with a subset of the best variables in order to refine the fit for those variables [57]. Variables with importance scores (see above) greater than the mean importance score were retained in the second pass. Model fit was evaluated using leave-one-year-out cross-validation [106]. The data set was split to omit a single year (and not merely a single observation). A new random forest model was then fitted and the model performance was evaluated for the omitted year. This was repeated for all years. The average skill score was then derived for the leave-one-year-out cross-validation approach. We then assessed the amount of variation uniquely explained by each variable retained in the model based on the cross-validation data set. We then attempted to refine the model further by removing all variables that uniquely contributed less than a threshold value (thresholds tried were 0, 0.001, 0.005, and 0.01) and re-fitting the random forest model. The model corresponding to the threshold that resulted in the highest cross-validated *R*^*2*^ was then retained and the reported final fit metrics correspond to these final models.

After running the model including all variables of interest, we repeated the analysis approach, but without the climate variables, to identify the degree to which climate variables uniquely contribute to the model fit (variance partitioning, [107,108]). We then repeated this analysis with only the climate variables, to assess the degree to which non-climatic variables influence the choice of climate variables included in the models.

For human case data, we first analyzed the data for the human subset (those counties for which we had both mosquito and human data). We constructed a random forest model to describe the relationships between human cases, the observed *MLE*, the climate covariates, and the other covariates, and partitioned the amount of variance due to each set of covariates [107,108], as was done for mosquitoes above. We then repeated the analysis for the human all counties data set, omitting the *MLE*, again partitioning the amount of variance due to each set of covariates. We note that human cases are discrete occurrences, but the model predictions were on a continuous scale. If this is of concern, we note that the model predictions could be rounded to the nearest integer value.

### Model fit statistics

#### Root Mean Squared Error (RMSE)

This was calculated with the RMSE function in the package caret in R [109]. Root mean squared error corresponds to the standard deviation of the residuals [105], and gives an estimate of the magnitude of the errors. It is expressed as number of infected mosquitoes per 1000 mosquitoes or number of human cases.

#### Median RMSE

RMSE was calculated for each year of the cross-validation data, and median RMSE corresponds to the median RMSE value from this evaluation. Median RMSE is less biased by a single extremely poor or extremely good prediction year.

#### Scaled RMSE

The Root Mean Squared Error was divided by the mean infection rate, to express the error as a percentage of the mean value. This has the benefit of placing it in the context of the values to be predicted, and may serve as a more intuitive measure of error. This quantity can vary from 0 to ∞, with 0 serving as no error, 1 indicating the model error is equal to the mean, and every value >1 indicating the number of times the error is greater than the mean.

#### Max error

The maximum error observed for any sample in the validation data set, expressed as number of infected mosquitoes per 1000 mosquitoes, or number of human cases.

#### Coefficient of determination (*R*^*2*^)

Here, *R*^*2*^ is defined by *Eq 5*:

$$R^{2}=1-\frac{\sum {\left(y-\hat{y}\right)}^{2}}{\sum {\left(y-\bar{y}\right)}^{2}}$$ (5)

Where *y* corresponds to the observed values in the validation set, $\hat{y}$ corresponds to the predicted values, and $\bar{y}$ corresponds to the mean of the validation set [105,110]. In contrast to computing an *R*^*2*^ for the data used to fit the model, where *R*^*2*^ is bounded between 0 and 1, by computing *R*^*2*^ for the validation data set, values can range from -∞ to 1. This occurs because it is possible for the model to have worse predictive power than the mean of the validation data set. A value of 1 would indicate a perfect fit, whereas a value of -1 would correspond to the model having twice the residual squared error of the validation data set’s mean value.

#### Spearman (*r*_*s*_) and Pearson (*r*_*p*_) correlation coefficients

These correspond to the Spearman correlation coefficient and the Pearson correlation coefficient, respectively, and were calculated with the cor function in Base R according to the standard formulae [59].

### Contour plot methods

We visualized the outputs of the random forest models by creating bivariate contour plots. Model predictions were generated for a regular grid of 100 points covering the parameter space of the two variables. Values for other covariates were fixed at their mean values. Contours were then drawn to indicated lines of equal predicted infection rates. Observed infection rates were overlaid as red circles, with the size proportional to infection rate. However, the observed and predicted infection rates are not strictly comparable, since the observed values are of course affected by state of the other covariates, rather than the mean covariate values. Nonetheless, the contour plots give some insight of the response function that are otherwise hidden in the high-dimensional nonlinear random forest regression model.

## Results

We present first a summary of the random forest model fitting results followed by results that address the question of which–if any—climate covariates improve the model skill. In the last part we describe the spatial and temporal variability for selected dependent and independent variables identified by the models as important.

**Fig 1:**
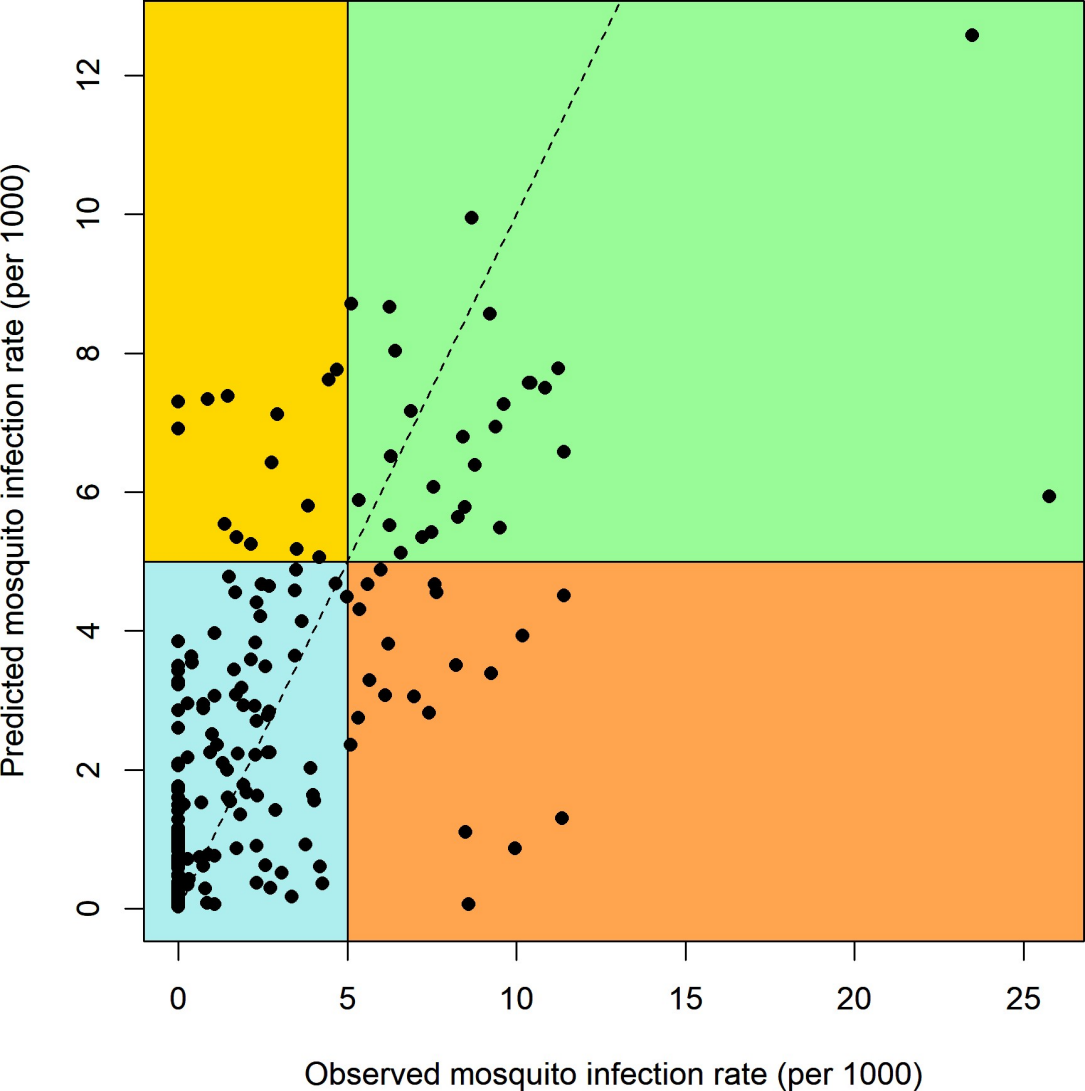
Observed mosquito infection rate (*MLE*) vs. predicted *MLE* from the WNV model using the entire data set. Background colors correspond to a classification of model predictions based on *MLE* of 5 [22]. Green corresponds to a correct prediction of high WNV *MLE* (27 records, 12.4%), blue corresponds to a correct prediction of low WNV *MLE* (157 records, 72.0%). Yellow corresponds to an error where the model predicts *MLE* to be high, but it is not (14 records, 6.4%), whereas orange corresponds to an error where the model predicts *MLE* to be low, but *MLE* was high (20 records, 9.2%). Future models should aim to improve the model’s sensitivity (0.57), although the specificity (0.92) is also of concern. Note that some predictions can be quite accurate, and still result in misclassification if they are near the classification threshold.

**Fig 2:**
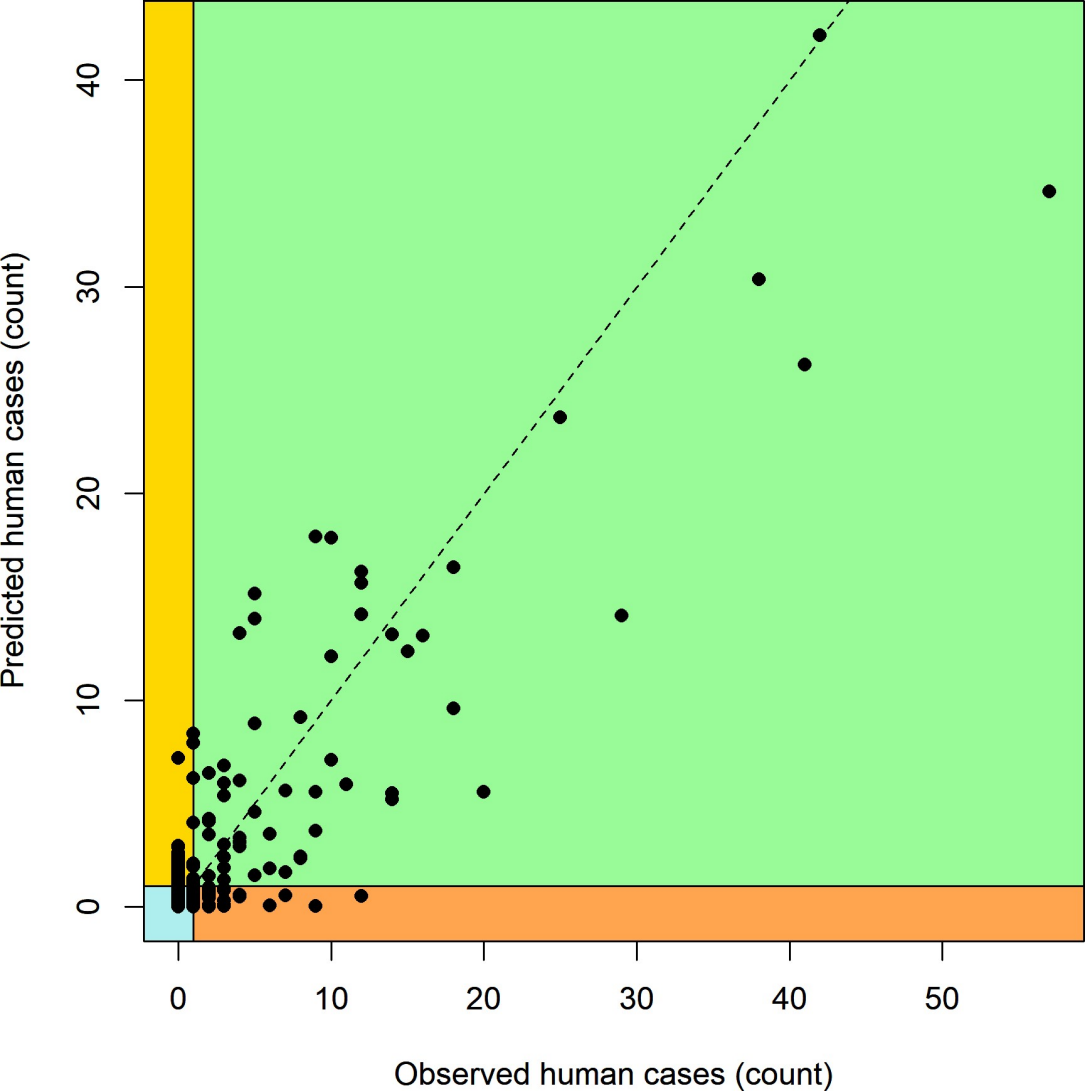
Observed number of human cases of WNV across all of New York and Connecticut vs. predicted number of human cases of WNV from the model using the entire data set. Background colors correspond to a classification of model predictions based on a threshold of 1 human case. Green corresponds to a correct prediction of one or more human cases (65 records, 7.4%), blue corresponds to a correct prediction of no human cases (704 records, 79.8%). Yellow corresponds to an error where the model predicts at least one human case, but none were observed (38 records, 4.3%), whereas orange corresponds to an error where the model predicts no human cases, but at least one was observed (75 records, 8.5%). Sensitivity (0.46) and specificity (0.95) were similar to the estimates for county-scale mosquito infection rates.

**Fig 3:**
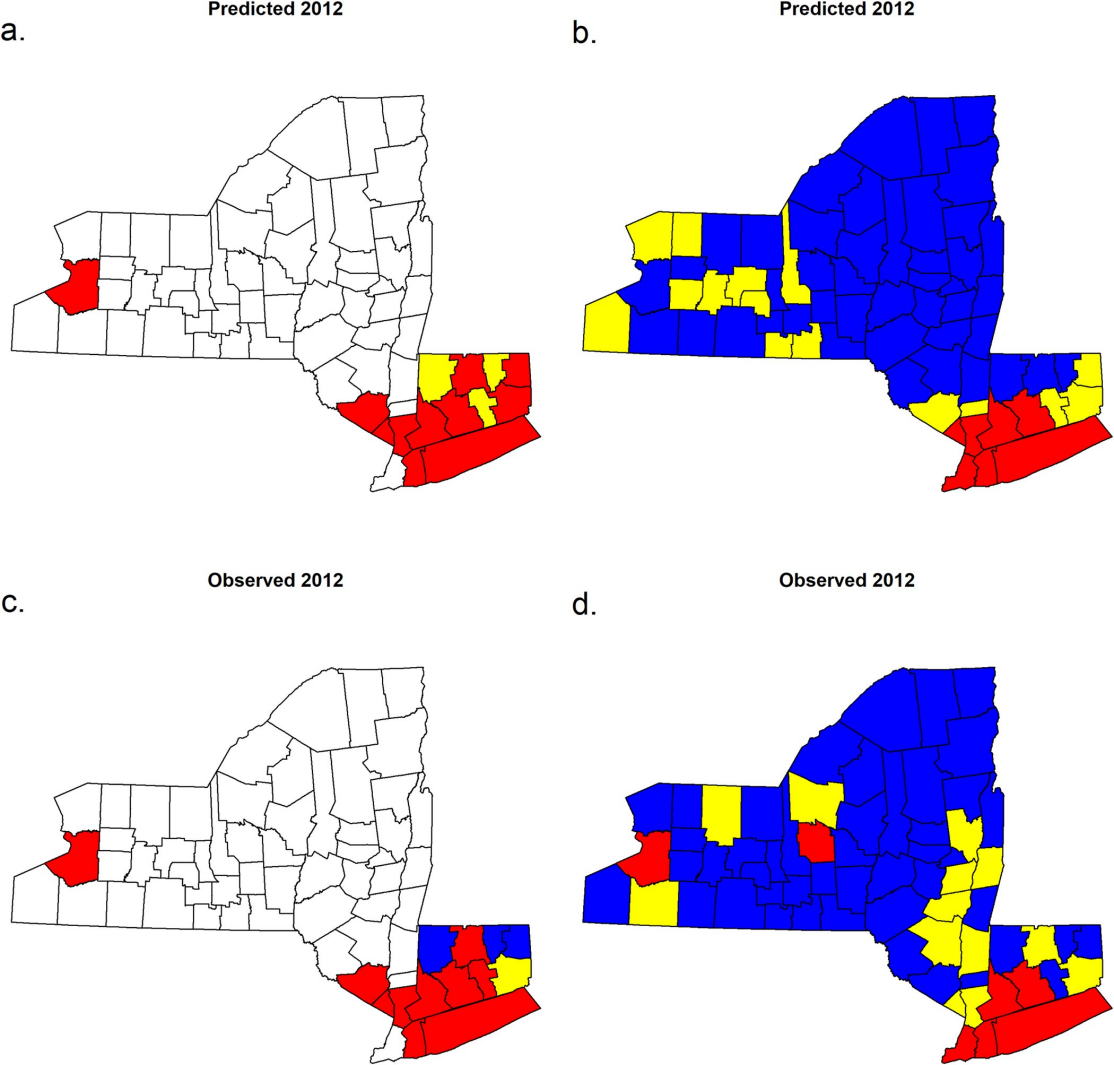
Predicted and observed WNV mosquito infection rates (*MLE*, a, c) and human cases (b, d) for 2012, a particularly widespread WNV year. *MLE* thresholds from Little et al. [22]: blue corresponds to *MLE* < 1 mosquito per 1000, yellow corresponds to *MLE* 1–5 per 1000, and red to *MLE* > 5 per 1000. White indicates excluded counties for which we did not have mosquito surveillance data. For human cases (b, d), blue indicates no human cases, yellow indicates 1–5 cases, and red indicates more than 5 cases.

**Fig 4:**
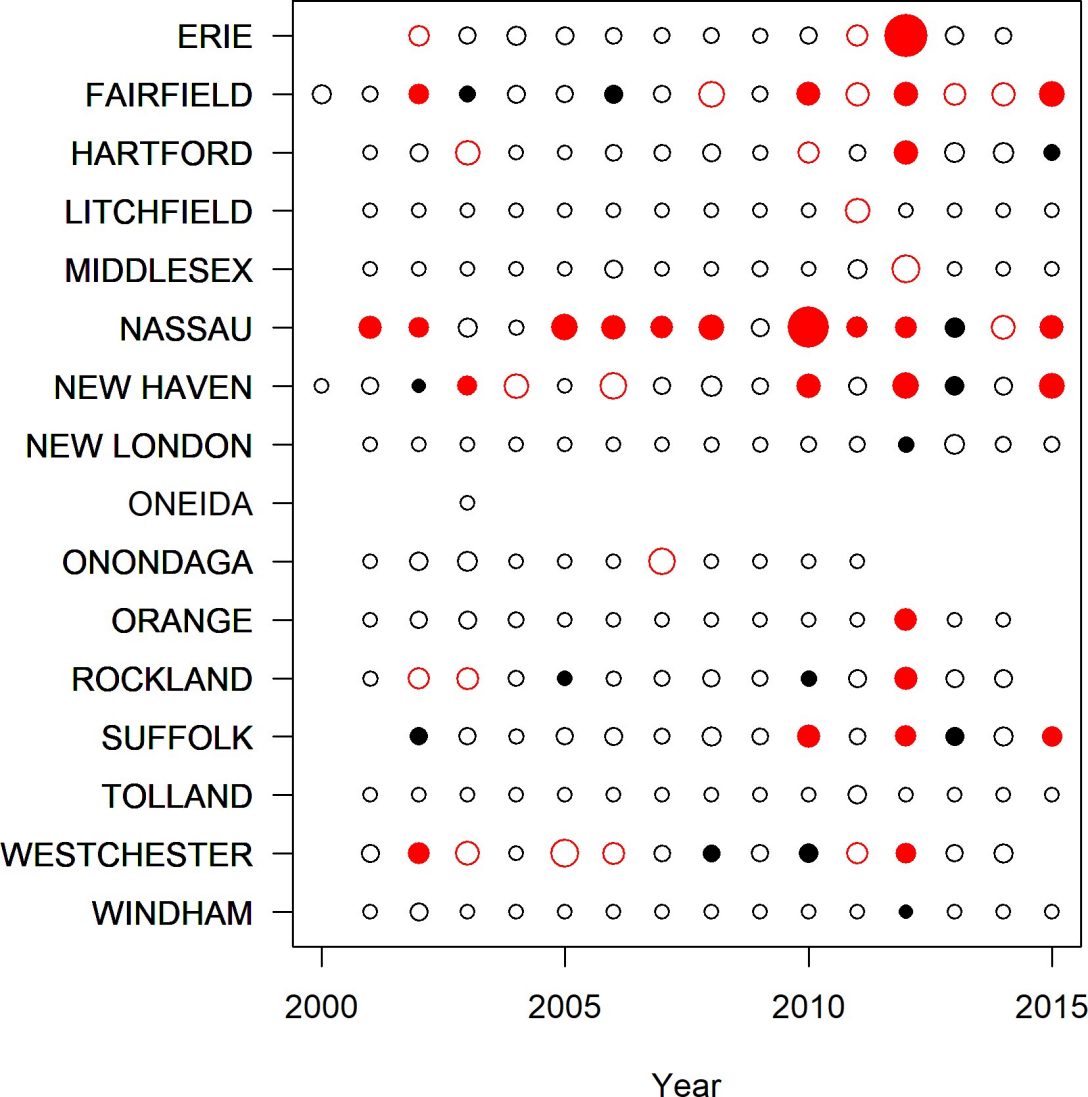
Predicted (unfilled ≤ 5, filled > 5) and observed (black ≤ 5, red > 5) infected mosquitoes per 1000 for each county and year for WNV. Missing points correspond to missing years for those counties. Point sizes are scaled relative to the observed infection rate.

**Fig 5:**
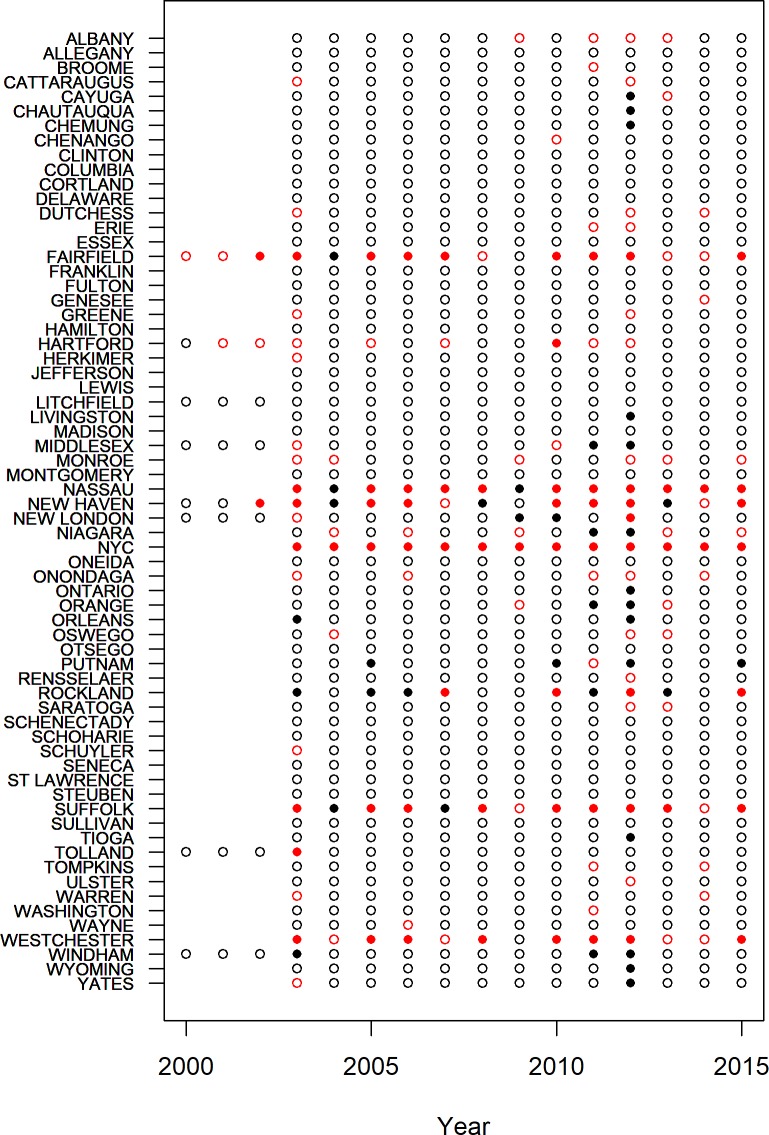
Predicted (open < 1, filled ≥ 1) and observed (black < 1, red ≥ 1) number of human WNV cases for each county and year. Data were not available for New York for 2000–2002, hence the missing points.

Our best-fitting model using the full data set at the county-scale explained 45% of the variation in *MLE* (Fig 1, Table 2), and 72% of the variation in human case counts (Fig 2, Table 3). When infection rates or case counts were converted to categories, the model both over- and under-predicted WNV risk for some locations and years (Figs 3–5). These results also highlight a few counties that have elevated *MLE* throughout the last 10–15 years (Fairfield, Nassau, New Haven, and Westchester), whereas others are still free of high *MLE*, as depicted in Fig 4 by the high number of red dots and black dots, respectively. These counties are also notable in the persistent accounts of reported human cases (Fig 5). Interestingly, Suffolk County showed high numbers of human cases over the years, whereas the WNV *MLE* are more temporally variable, likely due to our exclusion of light traps, which represent the majority of the trapping effort in this county. The year 2012 exhibits the highest number of counties with high *MLE* (10 of 14 counties with *MLE* > 5 infected per 1000). The model predicted 9 of those counties correctly, but underestimated risk in one county and overestimated it in 3 others (Fig 3). The years 2011 and 2013, before and after the peak year (2012) showed fewer counties with high *MLE*. The model generally reproduces region-wide variations, an indication that climatic conditions play a role in the WNV *MLE*.

**Table 2:** Model fit results for the calculated mosquito infection rates (per 1000). Climate indicates whether climate variables were included, *N* indicates sample size, while WNV+ N indicates the number of samples estimated to have WNV present. RMSE, Median RMSE, Max Error, Scaled RMSE, *R*^*2*^, *r*_*p*_, and *r*_*s*_ are defined in *Methods*: *model fit statistics*.

Scale	Climate	*N*	WNV+ *N*	RMSE	Median RMSE	Scaled RMSE	Max Error	*R*^*2*^	*r*_*s*_	*r*_*p*_
County	YES	218	132	2.8	2.3	1.04	19.8	0.45	0.69	0.68
County	NO	218	132	3.3	2.7	1.21	23.3	0.26	0.67	0.51
Trap	YES	3156	955	8.2	7.7	2.34	87.4	0.16	0.45	0.40
Trap subset	YES	2596	395	1.2	1.0	2.13	15.4	0.53	0.59	0.73
Trap subset	NO	2596	395	1.4	1.2	2.49	10.9	0.36	0.57	0.61

**Table 3:** Model fits for the human data at the county-scale. The All Counties analysis was based on 882 county × year records, while the subset contained 206 county × year records for which surveillance data were available. RMSE, Max Error, Median RMSE, Scaled RMSE, *R*^*2*^, *r*_*p*_, and *r*_*s*_ are defined in *Methods*: *model fit statistics*.

Scale	Climate	RMSE	Median RMSE	Scaled RMSE	Max Error	*R*^*2*^	*r*_*s*_	*r*_*p*_
All Counties	YES	2.0	1.6	2.45	30.2	0.72	0.39	0.86
All Counties	NO	2.5	1.7	2.93	37.6	0.60	0.45	0.79
Subset	YES	3.7	1.7	1.80	42.3	0.52	0.70	0.72
Subset	NO	4.0	2.1	1.94	44.1	0.45	0.66	0.68
Subset -S^1^	YES	3.9	1.7	1.88	43.1	0.48	0.70	0.69

^1^ Without surveillance variables

**Fig 6:**
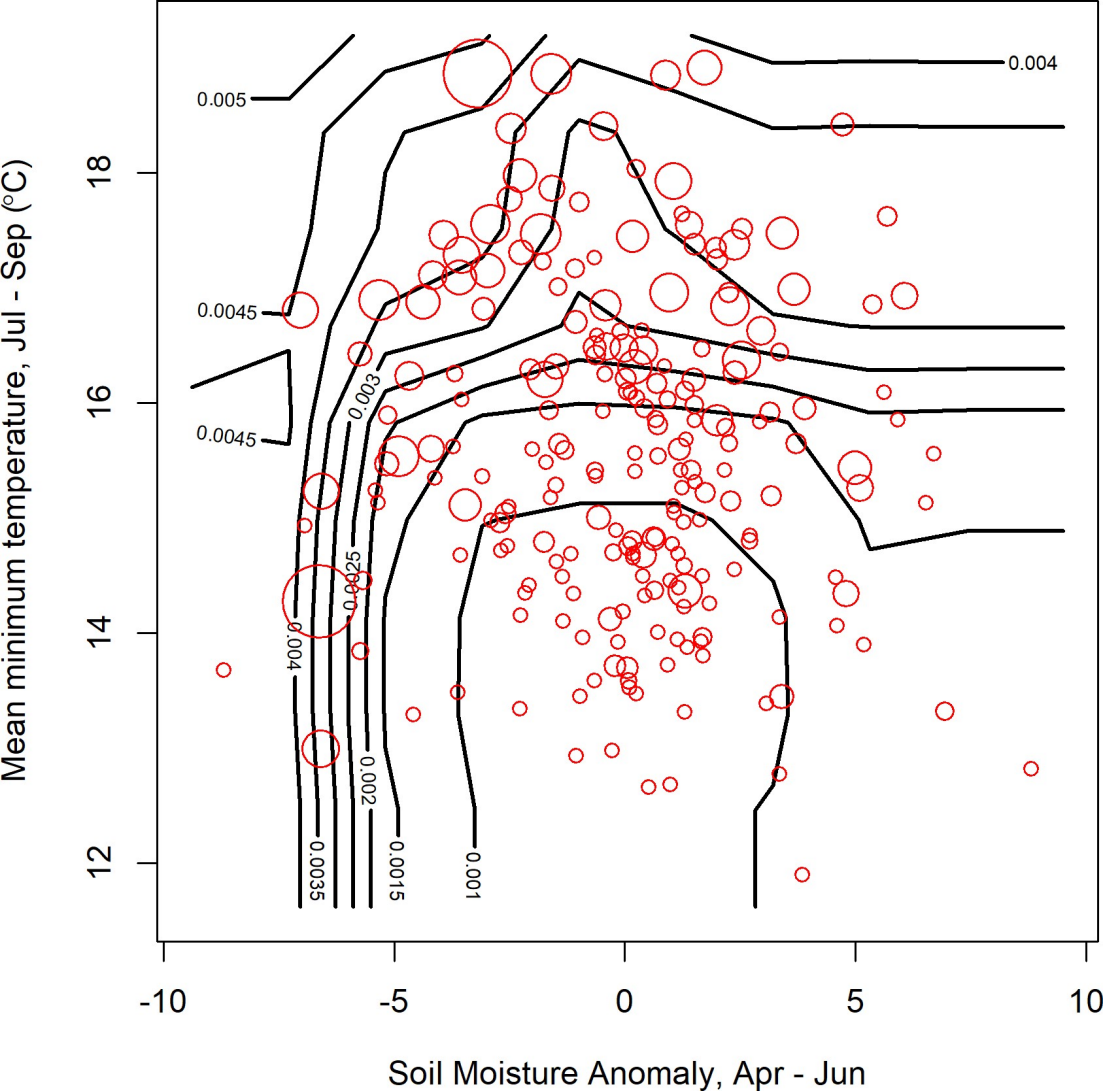
Predicted mosquito infection rates (*MLE*, contours) increase non-linearly with 2^nd^ quarter soil moisture anomaly and 3^rd^ quarter temperature. Cool years with normal soil moisture were associated with the lowest *MLE*. Warm years showed high *MLE* regardless of soil moisture and dry years often (but not always) had high *MLE*. Observations (red circles, size is proportional to *MLE*) broadly support these predictions. Contour lines correspond to predictions made for a regular grid of 100 points covering the range of both variables. Predictions were made for mean values for all other covariates (see Tables 4 and 5 for included variables, see S1 File for mean values), while observed values correspond to the exact variable combinations and therefore may not exactly correspond to the predictions. Observations are plotted as a general guide to identify major patterns and highlight particular exceptions.

**Fig 7:**
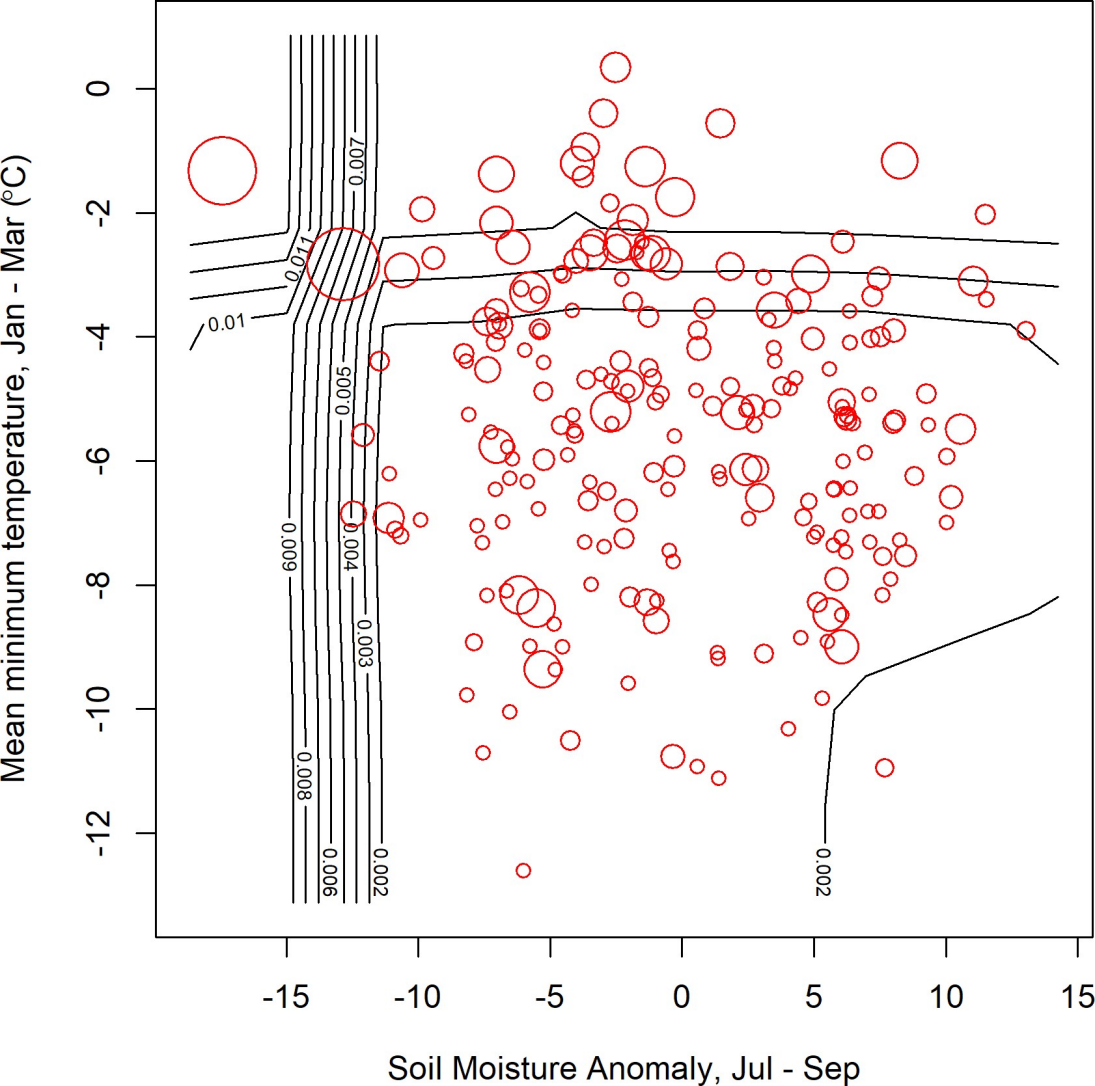
Warm winter temperatures and dry summers were associated with the highest risk of mosquito infection with WNV. Observations (red circles, size is proportional to infection rate) broadly support these predictions. Contour lines correspond to predictions made for a regular grid of 100 points covering the range of both variables. Predictions were made for mean values for all other covariates (see Tables 4 and 5 for included variables, see S1 File for mean values), while observed values correspond to the exact variable combinations and therefore may not exactly correspond to the predictions. Observations are plotted as a general guide to identify major patterns and highlight particular exceptions.

**Fig 8:**
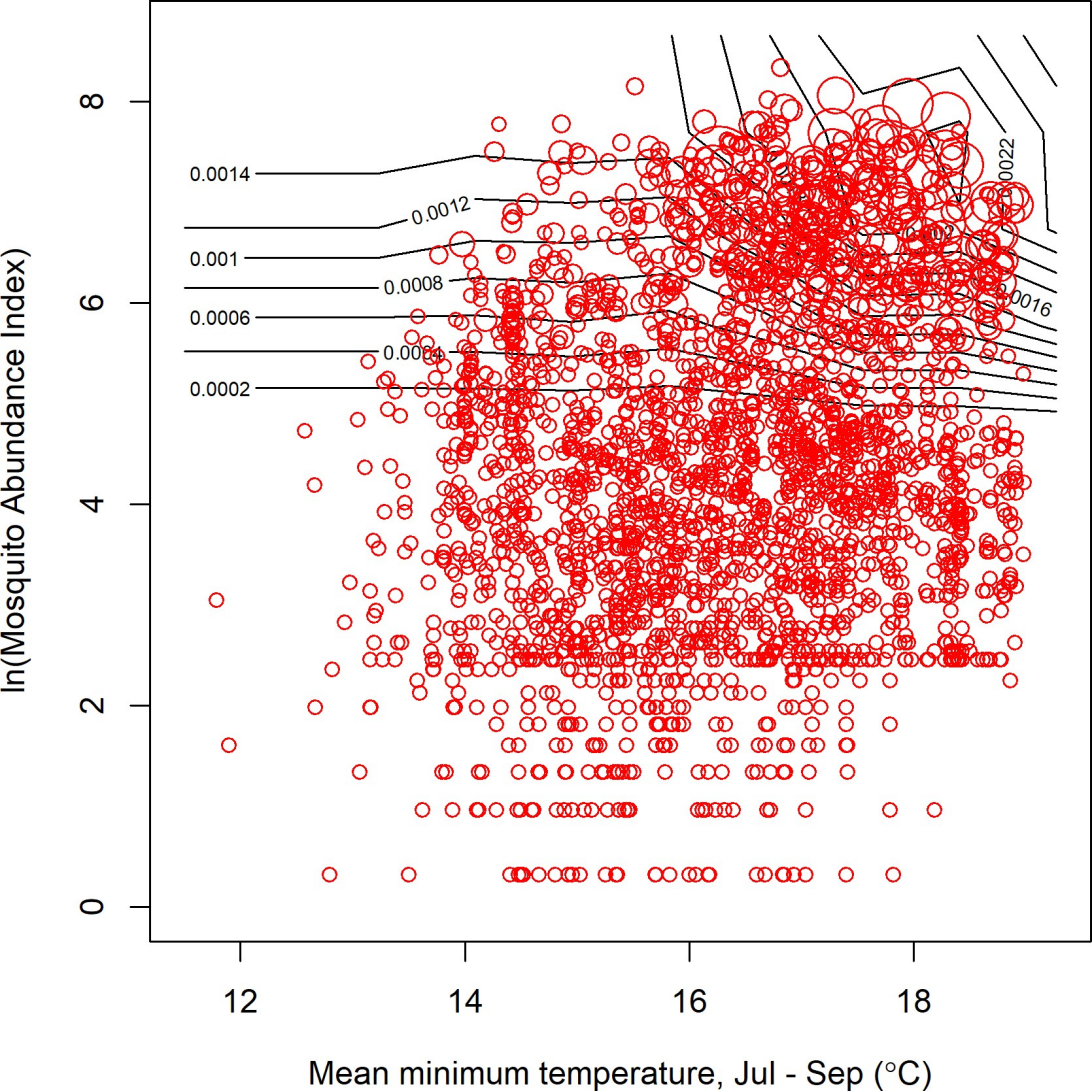
For individual trap sites, the risk of WNV increased with increasing mosquito abundance, especially when the mean minimum temperature in the 3^rd^ quarter was high. Contour lines correspond to predictions from a regular grid of 100 points, (with values from other covariates fixed at a mean value). Observed infection rates (red circles, size is proportional to infection rate) are plotted for comparison, but note that they use exact parameter combinations and not the mean conditions used for making the predictions.

**Fig 9:**
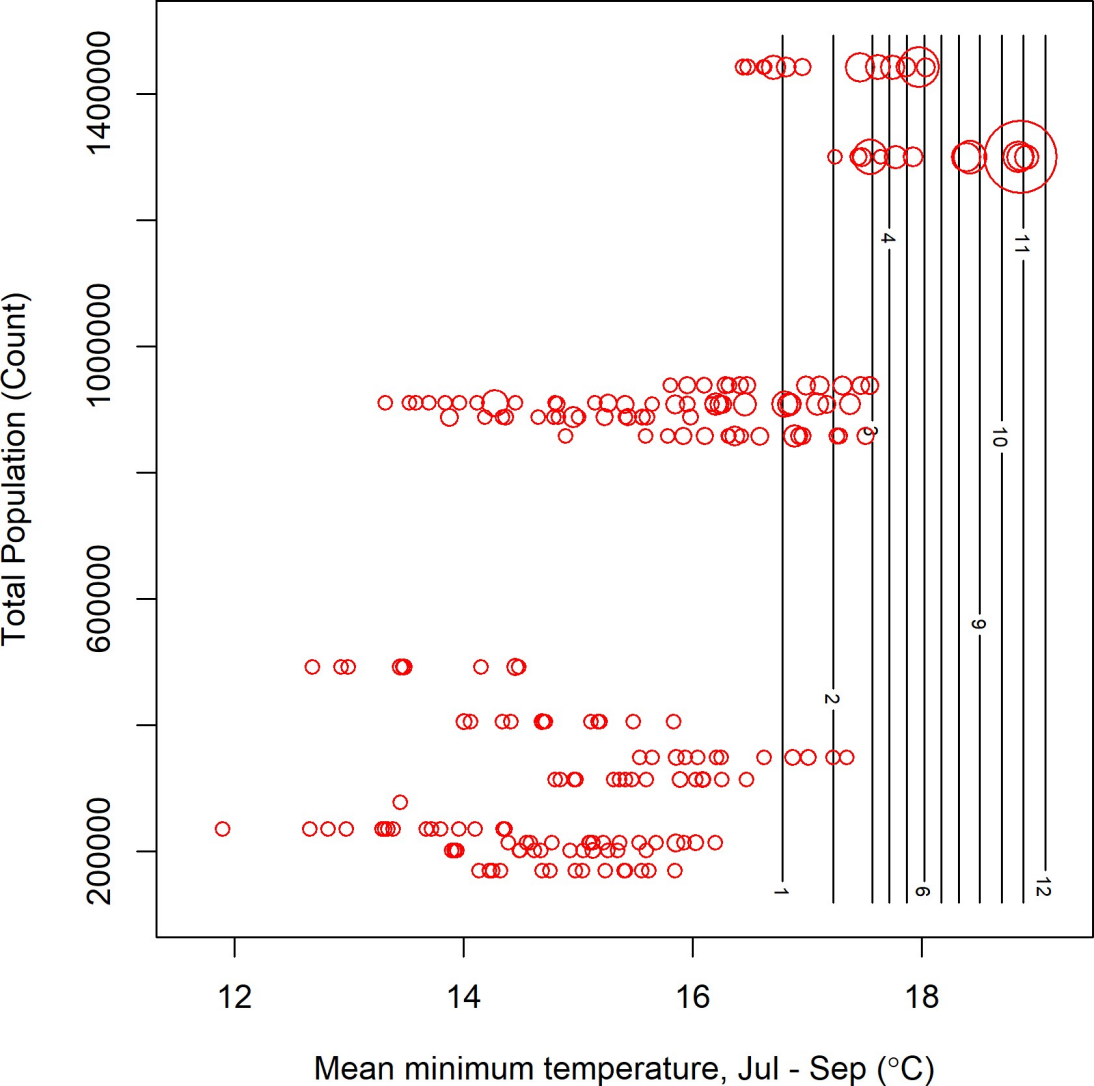
Risk of human cases of West Nile were highest for locations with high total populations, especially in years with a warm summer. Data correspond to the human subset analysis. Contour lines correspond to predictions from a regular grid of 100 points, (with values from other covariates fixed at a mean value). Observed infection rates (red circles, size is proportional to infection rate) are plotted for comparison, but note that they use exact parameter combinations and not the mean conditions used for making the predictions.

The accuracy of predictions was variable for individual counties or years (Figs 4 and 5). The models retained both climatic and non-climatic variables in the final predictive models (Tables 4 and 5). In particular, *MLE* increased with increasing mean minimum temperature for July, August and September (Table 4, Fig 6). At the county scale, *MLE* showed a non-linear relationship to soil moisture in April, May and June. Years with low soil moisture were always at risk of high WNV, years with normal soil moisture corresponded to a risk of WNV when mean minimum temperatures were high, and years with above-normal soil moisture were associated with a slight increase in WNV risk relative to years with normal soil moisture and cool mean minimum temperatures (Fig 6). High mean minimum temperature in January, February and March was also associated with higher WNV rates, as were droughts in July, August and September (Fig 7). At the scale of individual traps, mosquito abundance and maximum observed temperature from April to June were among the most important variables (Table 4, Fig 8). American Robin abundance was also predictive of mosquito infection rates. Similar to the mosquitoes, human cases also show an increase with mean minimum temperature for July, August and September, and with total human population in the county (Fig 9).

**Table 4:** Climate variables identified as important by the random forest model when the model with all covariates was run, and when a model with only climate covariates was run (only C). Model results are presented for human cases in those counties where mosquito surveillance data were collected, and for mosquito infection rates (*MLE*) at both the county and trap scales. Values in the table indicate the amount of unique variation explained by the variable using variance partitioning, while a blank indicates that the variable was not included in the final predictive model.

Variables appearing in a final model	Human subset	Human subset only C	MLE county	MLE county only C	MLE Trap subset	MLE Trap subset only C
Mean minimum temperature (Jan–Mar)		<0.001	0.01	0.001		
Mean minimum temperature (Apr–Jun)						0.01
Mean minimum temperature (Jul–Sep)	0.004	0.03	0.01	0.02	0.01	0.01
Mean minimum temperature anomaly (Oct–Dec)						0.01^a^
Mean maximum temperature (Jan–Mar)			0.003	0.001		0.01
Mean maximum temperature (Jul–Sep)			0.001			
Mean maximum temperature anomaly (Jan–Mar)			0.002			
Minimum observed temperature (Jul–Sep)		0.01				
Minimum observed temperature (Oct–Dec)						0.01
Maximum observed temperature (Apr–Jun)	0.02	0.01			0.03	
Maximum observed temperature (Oct–Dec)				0.02^a^		
Maximum observed temperature anomaly (Apr–Jun)	0.02	0.01				
Daily temperature range (Jan–Mar)		0.003				
Daily temperature range (Jul–Sep)						0.01
Daily temperature range (Oct–Dec)				0.004^a^		
Daily temperature range anomaly (Jan–Mar)						0.02
Soil moisture anomaly (Apr–Jun)			0.03	0.04		
Soil moisture anomaly (Jul–Sep)			0.04	0.05		
Soil moisture anomaly (Oct–Dec)						0.01^a^
Growing degree days (Jul–Sep)		0.002				
Growing degree days anomaly (Apr–Jun)						0.01
Growing degree days anomaly (Oct–Dec)	0.01^a^	0.02^a^				0.01^a^

^a^ We hypothesize that the contribution of this variable is related to the end of the mosquito season in October.

**Table 5:** Non-climatic variables identified as important by the random forest model when the model with all covariates was run, and when a model without climate covariates was run (-C). Model results are presented for human cases in those counties where mosquito surveillance data were collected, and for mosquito infection rates (MLE) at both the county and trap scales. Values in the table indicate the amount of unique variation explained by the variable using variance partitioning.

Variables appearing in a final model	Human subset	Human subset -C	MLE County	MLE County -C	MLE Trap	MLE Trap -C
Mosquito infection rate	0.02	0.05	NA	NA	NA	NA
Mosquito abundance index					0.15	0.28^a^
Mosquito density index						0.06^a^
Trap bait type						
Total population	0.01	0.02	0.003	<0.001		
Population density		0.02		0.002	0.01	
Percent urban		0.02		<0.001	0.01	
Percent forest	0.002	0.03		<0.001		
Percent open		0.02		0.001		
Percent wetland				0.002		
American Robin Index		0.06		0.02	0.01	0.02^a^
American Crow Index		0.002		0.01		0.03^a^

^a^ We note that the sum of the values in this column exceeds the total amount of variation explained by the model (0.36). This occurred because the model without one or more of these variables explained less variation than just using the mean value from the validation data set and therefore had a negative *R*^*2*^ value as the baseline instead of zero (see *Coefficient of determination* section in methods for the method of calculating the *R*^*2*^).

### Model fit with and without climate variables

Models without climate variables explained 17–19% less of the total variance in *MLE* than models with climate variables (Table 2) and removal of climate variables resulted in a poorer fit (Table 2). Although the mean errors were smaller with climate variables included, the maximum error observed was sometimes greater (i.e., when evaluated at the trap-subset scale). Removal of climate variables led to changes in which non-climatic variables were included in the models (Table 4). Models that include climate variables explained 7–12% more of the total variance for the number of human cases (Table 3). Again, removal of climate variables led to the inclusion of additional non-climatic variables into the model (Table 5). For both *MLE* and human cases, Pearson correlation coefficients were higher with climate variables included (Δ*r*_*p*_ 0.12–0.17 for *MLE*, 0.04–0.07 for human cases, Tables 2 and 3), whereas Spearman correlations were often similar with and without climate variables (Δ*r*_*s*_ 0.02 for *MLE*, -0.06–0.04 for human cases, Tables 2 and 3). This suggests that climate variables improve the estimation of the magnitude of WNV outbreaks across years; in contrast non-climatic variables may determine the baseline risk for a given location.

### Model fit by scale

The trap-scale subset had the lowest RMSE for *MLE*, whereas the trap-scale had the highest RMSE (Table 2). The county-scale had the lowest RMSE when scaled by the mean infection rate. The percent of variation in *MLE* explained relative to the mean (*R*^*2*^) was also greatest at the trap-scale subset (Table 2).

### Description of key variables

**Fig 10:**
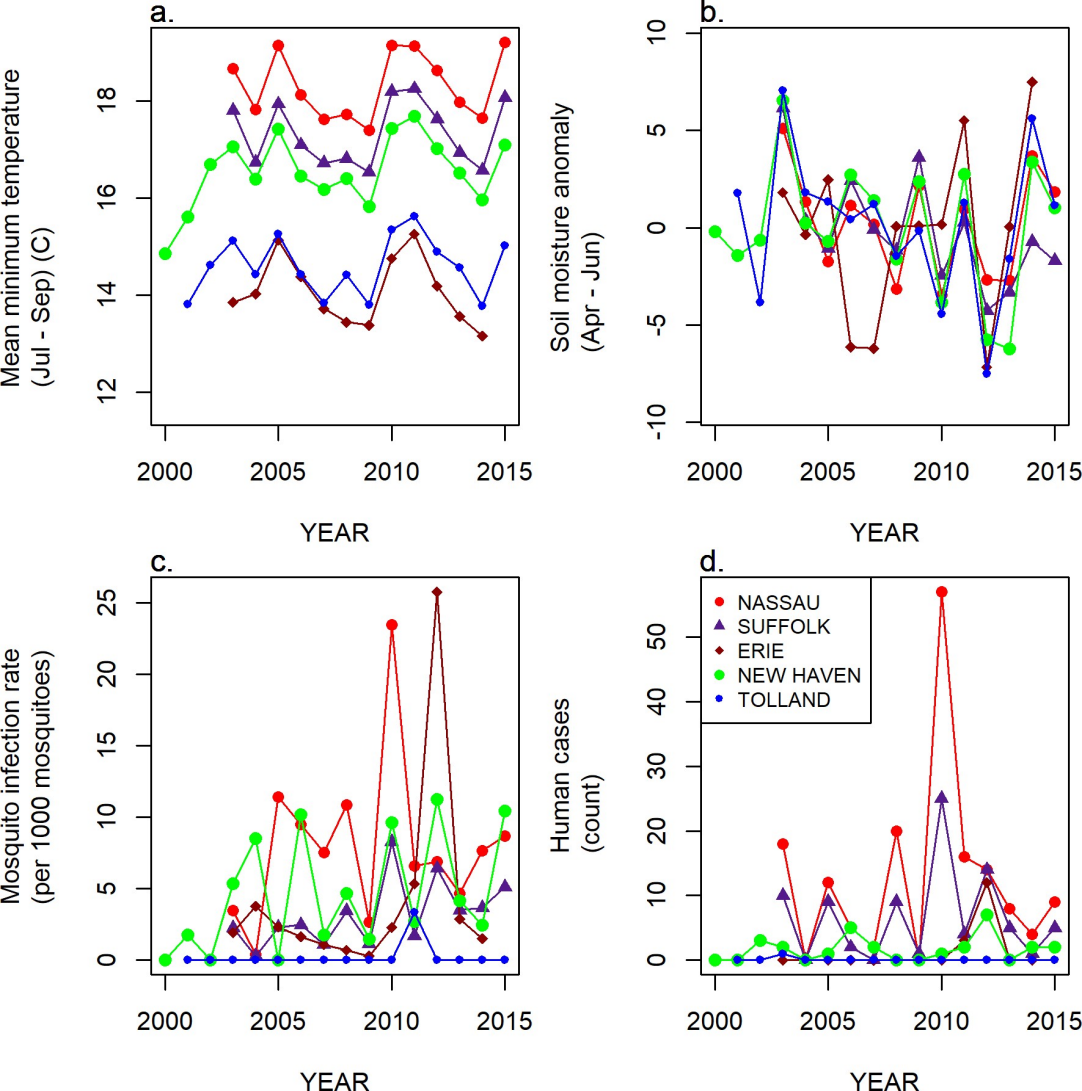
Mean minimum temperature (a), soil moisture anomaly (b), mosquito infection rate (c), and human case counts (d) by year for five example counties.

**Fig 11:**
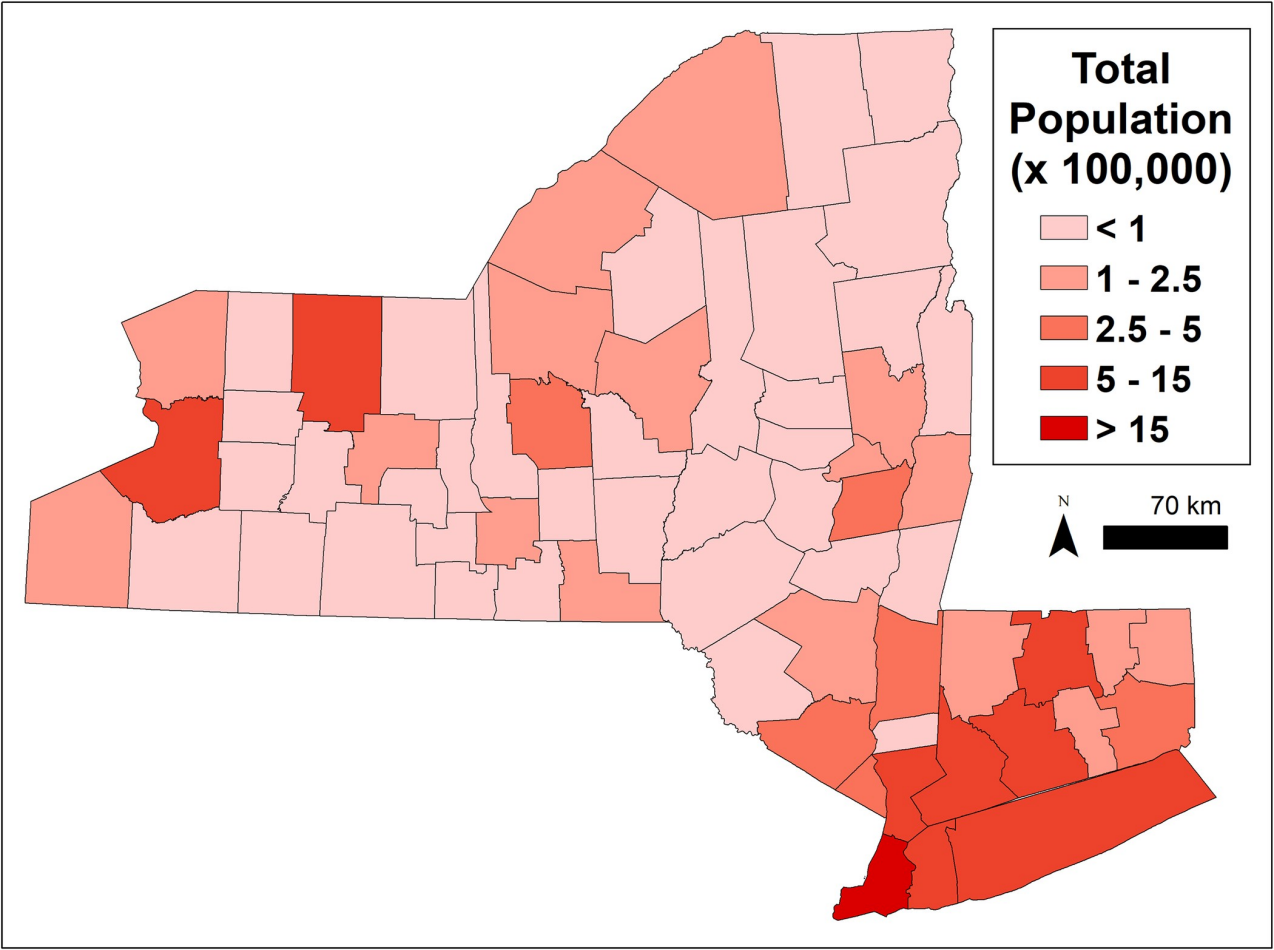
Total human population of the study region. Note that the five counties of New York City have been merged into a single entity. Data taken from the US Census [100,111].

Mean minimum temperatures (3^rd^ quarter) were highly temporally correlated across counties (Fig 10A), although counties differed in their mean temperature. Soil moisture anomalies (2^nd^ quarter) were also correlated, but showed some large, county-specific deviations (Fig 10B). Human cases often, but not always, tracked mosquito infection rates (Fig 10C and 10D). Total population was also identified as important by many of the models (Table 4), and the population distribution is presented in Fig 11.

## Discussion

We found that climate variables improved WNV model fit metrics at both coarse and fine scales (with a few minor exceptions, see Tables 2 and 3). Climate variables were especially important for predicting human West Nile cases across all counties (ΔR^2^ = 0.12), and mosquito *MLE* at the county- and trap-scales (ΔR^2^ = 0.19, ΔR^2^ = 0.17, respectively). We found evidence that some of the climate effects on WNV were an indirect result of climatic effects on mosquito populations (see e.g., [112]). When climate data were omitted, *MLE* became more important in predicting human cases and the mosquito abundance index became more important for explaining *MLE* at the trap scale (Table 5).

Within the climate predictor set, the mean minimum temperature in July, August and September was frequently included as a predictor in the best models. Human population was also consistently important in predicting both *MLE* and number of human cases (Table 5), consistent with the urban nature of *Cx*. *pipiens* and results of prior research (e.g., [39]. When climate variables were excluded, the importance of mosquito infection rate for predicting human cases greatly increased. This further supports a major role of climate mediating mosquito infection rates.

Our results are broadly consistent with nine previous studies that have included climate data and NY or CT in their scope (Table 1). To some degree, this was due to analyzing data that was also incorporated in the prior studies. For the analyses across the United States, our human case data represent a subset of their overall data set. In contrast, the data used in several more localized studies in CT, Suffolk County, and Erie County represent subsets of our larger data set. To our knowledge, the mosquito surveillance data set used here represents the largest data set applied to NY and CT at the county scale or below. Our study also differed from most previous analyses in this region (except [55]) in our use of machine learning techniques.

We found that the RMSE estimates produced by the models were highly scale-dependent. As the spatial scale changed, the estimated infection rates changed. For the trap-scale subset, this is due in part to dropping records with high uncertainty in the mean estimate. This disproportionately, but not exclusively, affected sites where WNV was detected. Second, the difference in estimated infection rates could be due to aggregating samples in the presence of spatial heterogeneity and unequal sampling [33]. This scale-dependence of fit statistics is important to consider when comparing results across studies, and highlights the value of standardizing the RMSE by the mean infection rate. Otherwise, one might conclude unequivocally that the trap-scale subset results were more accurate based on the RMSE. Although in terms of absolute error this is true, the lower mean infection rate indicates that there was less potential for error in that model and the scaled RMSE indicates greater error relative to the mean value. More broadly, while our model RMSE of 2.8 at the county-scale is similar to the RMSE of 4.3 observed by Little et al. [22] for Suffolk County, we note that the results are not strictly comparable as their model was evaluated at a 13 × 13 km scale, in contrast to our results that were at the county-scale, and no scaled RMSE was reported for that study. For humans, the RMSE of 2.0 indicates that our model predictions were off by an average of ±2 human cases. This number must be interpreted with caution, though. We speculate that much of this error was due to a few years with exceptionally high numbers of cases (maximum error of 30.2), as the median RMSE was 1.6 and squared errors are especially sensitive to large deviations (Table 3, All Counties results).

Here, we created predictive models with a minimum number of variables (minimum predictive models). It is worth noting that our variable selection approach did not identify all relevant covariates [113,114]. For example, changing the random forest starting seed changed which variables were included in the final model (not shown). This is due to two issues 1) collinearity among predictor variables (e.g., Fig 9) and 2) the complexity of the system (e.g., Fig 6). The high correlation among some of the variables (S7 File) may have obscured which variable(s) were the most important from a mechanistic standpoint. As Fig 9 shows, total human population and minimum 3^rd^ quarter temperature are both potentially important for explaining observed infection rates, but the range of variation makes it difficult to separate the individual effects of these variables. This is further complicated by the complexity of the system. Fig 6 demonstrates the potential for interactions between variables, for example, where the relationship with soil moisture depends on the temperature. Above a certain minimum temperature, there is no longer a strong relationship with soil moisture. There are many possible interactions among variables, and conclusively identifying them with small data sets may not be possible. We note that this issue is not unique to the random forest approach employed here. Little et al. [22], using linear methods, also found that multiple models with very different sets of variables had similar explanatory power for predicting WNV.

It is interesting to note, however, that the model predictions were very good when extreme climate conditions were encountered. Exceptionally warm average minimum temperatures in January–March and from July–September, were often associated with a much higher risk of WNV. Drought conditions reduced the minimum temperatures necessary for a WNV outbreak, or may have amplified the magnitude of the outbreak. In contrast, during the typical minimum temperature or during normal soil moisture, WNV risk was reduced, but more difficult to predict precisely, due to variation in *MLE* when climatic conditions were similar.

### Limitations of the present study

Future research could include additional covariates, such as multiple buffer distances at the trap-scale [46], the Normalized Vegetation Difference Index [43,46], socio-economic variables (especially age) [52,115–117], changes in host behavior (related to a shift in mosquito feeding preferences) [20], rates of host immunity, rate of human immunity [31], degree of *Cx*. *pipiens pipiens* × *Cx*. *pipiens molestus* hybridization due to changes in contact rates [74], mosquito control activities and lagged climate effects [28,29]. Of the omitted covariates, human age, mosquito control activities, and climate-lags may be the most critical. Age is a major factor in whether WNV becomes neuroinvasive [1,118], and the number of susceptible humans could be an important consideration [31]. However, one study found reduced WNV in areas with elderly populations due to those populations being located in areas that were less risky for WNV based on degree of urbanization [52]. Mosquito control efforts could contribute to a mismatch between predictions and observations. If conditions are suitable for WNV, but mosquitoes have been controlled, the model may predict high WNV risk, but the actual risk may be low. Conversely, if mosquito control is included in the training data set, predictions for areas where control is absent but risk is otherwise high, could be low. These variables have been difficult to include: to our knowledge, one study included detailed information on the number of mosquito complaints and number of known larval sites [52], but none have included detailed spatial information of mosquito control activities. We suggest that such a data set would be highly beneficial. Our study, in contrast to others (e.g., [28,29]), did not consider lagged climate effects. In particular, prior-year precipitation has been found to influence WNV [29]. However, we note that several of the variables identified here would be available by early April or early July, and therefore could provide some predictive skill prior to the onset of human West Nile cases.

Statistically, the methods employed here could be further refined. Spatial and temporal autocorrelation may substantially influence model results [119,120]. We did not detect evidence of temporal or spatial autocorrelation based on a visual inspection of the model residuals [55]. It is possible that more refined models with respect to spatial or temporal autocorrelation could result in further improvements to the model fit statistics. However, we believe the process of evaluating model fit based on a validation data set indicates that our results are not a simple result of autocorrelation. Additionally, a prior study found no benefit to including a spatial autoregressive coefficient [25], but see [42].

Spearman correlations were not very different between the climate and non-climate models. One contributing factor may be the difficulty in obtaining unbiased results from rank order correlation statistics in the presence of zero-inflated data [121]. When WNV is absent, it creates a multi-way tie for the last rank. In contrast, the random forest model generated continuous estimates of WNV risk, making predicted ties unlikely. Consequently, a model could have a very low absolute error but still have a low Spearman correlation in the presence of zero-inflated data.

Some of the methodological decisions made in this study may also have influenced the lack of model fit. We restricted our analysis to gravid traps, and this decision under-sampled *Cx*. *salinarius*, as this species is trapped in greater numbers at light traps. In addition, other researchers have stated that gravid counts can be negatively affected when there are other sources of stagnant water [28], providing a possible bias towards fewer mosquitoes collected when there is higher precipitation. Trap success may also depend on the “pungency” of the trap water [28]. We considered only *Cx*. *pipiens*, *Cx*. *restuans*, and *Cx*. *salinarius* pooled together, as these three species were responsible for the majority of WNV positive pools in our data set, although 33 mosquito species in the Northeast [122] and at least 59 species worldwide have tested positive for WNV [64]. Importantly, the biology of *Cx*. *pipiens*, *Cx*. *restuans*, and *Cx*. *salinarius* differ, and pooling them may have increased the variation in our study. Methods have been developed to integrate multiple mosquito species into a single model [12], however this approach requires information on mosquito feeding preferences, which can vary spatially and temporally, even within a species [20,70]. It is possible that an analysis that spanned the entire mosquito community could improve the prediction of WNV in humans and mosquito pools. An additional decision was to use the climate from the centroid of each county for the county-scale. Visual inspection suggested that our climate data were similar across counties (we compare the centroid to the county average in S6 File). The lack of standardization in the methods used to collect the mosquito data (S5 File) may have increased the variance associated with sampling error, and thereby contributed to the remaining unexplained variation. For example, trap sites in three CT counties were primarily urban/suburban, whereas those in the remaining five counties were primarily rural and sparsely populated. Sampling in NY was not independent of the presence of WNV, and this may have biased our results. The precise timing of sampling varied from county to county, and from year to year (S5 File). This could potentially bias the results, as more sampling outside the peak WNV season is expected to lead to lower overall annual *MLE*.

### Future directions

Climate data from programs such as the Subseasonal to Seasonal (S2S) prediction project [123] and Subseasonal Experiment (SubX) provide novel opportunities for developing predictive models for WNV prevalence. Aside from the inherent uncertainties in seasonal climatic predictions, the success of seasonal predictive tools targeting infectious diseases such as WNV will ultimately depend on the robustness of the connecting links between climate and the targeted biological-epidemiological system.

### Conclusion

The WNV model developed here demonstrated predictive skill at multiple spatial scales for mosquito infection rates and human West Nile cases (see *R*^*2*^ values, Tables 2 and 3). Including climate data improved model predictions substantially, as evidenced by the ability to explain a higher fraction of the total variance in the validation data. The applied random forest model appears to provide a valuable and highly adaptable statistical tool for the prediction of infectious spatial and temporal disease. However, it must be emphasized that more research is needed to improve the understanding of the mechanistic processes.

One of the remaining challenges is to deploy the model predictions in decision-making processes. Prediction errors could lead to costly action (in terms of time and money) when no increased WNV risk is present, or costly inaction (in terms of human and ecological health) when an increased WNV risk is present but not predicted by the model. Model errors, as demonstrated by the maximum errors, were sometimes substantial, although this may in part reflect the uncertainty in the estimated mean infection rates for the sampling units. Therefore, improved models likely require further refinement to be useful in an operational context. However, the model has heuristic value in helping to understand the dynamics of WNV and may be useful when extreme climatic conditions are present and risks of WNV are straightforward.

## Supporting information

S1 File Descriptive Statistics (.zip containing .csv files): Mean, standard deviation, median, minimum, maximum, the median, minimum, and maximum range observed within years, the median, minimum and maximum range observed across different years for variables included in the model. (1_1 for county_annual_mosquito, 1_2 for county_annual_human, 1_3 for point_annual_mosquito).
(ZIP)

S2 File Data Dictionary for variable names. (CSV)

S3 File Data used to run the models at the county scales (.zip containing .csv files, see Data dictionary for variable names). S3_1 for county_annual_mosqutioes, S3_2 for county_annual_human, and S3_3 for county_annual_human_subset.
(ZIP)

S4 File A comparison of the model results for incidence and total cases. (DOCX)

S5 File Mosquito sampling methods. (DOCX)

S6 File Comparison of climate data based on centroid and based on an average. (DOCX)

S7 File Correlations by scale (.zip containing .csv files): Bivariate correlations (Pearson) between all the dependent and independent variables used in this study for each spatial scale (S7_1 for county_annual_mosquitoes, S7_2 for county_annual_human, S7_3 for county_annual_human_subset, S7_4 for trap_annual_mosquitoes for all trap sites, S7_5 for trap_annual_mosquitoes for the high-quality subset of trap sites). (ZIP)
