# Supplementary material for: Seasonal temperatures and hydrological conditions improve the prediction of West Nile virus infection rates in Culex mosquitoes and human case counts in New York and Connecticut
Source: PLoS One. 2019 Jun 3;14(6):e0217854. doi: 10.1371/journal.pone.0217854 (PMC6546252; doi:10.1371/journal.pone.0217854)
Supplement: S4 File — (DOCX) [file pone.0217854.s004.docx]

**Supplement S4 for:**

**Seasonal temperatures and hydrological conditions improve the prediction of West Nile virus infection rates in *Culex* mosquitoes and human case counts in New York and Connecticut**

Keyel, A.C., Elison Timm, O., Backenson, P.B., Prussing, C., Quinones, S., McDonough, K., Vuille, M., Conn, J.E., Armstrong, P.M., Andreadis, T.G., and Kramer, L.

**A comparison of the model results for incidence and total cases**

Incidence reduced the quality of fit for the analysis based on all counties, and provided a very similar level of fit for the subset of counties with surveillance data (Table S4.1). The incidence analysis selected some different variables (Table S4.2) than were identified based on total number of cases (Table 4). The relevance of these differences is unclear, as our statistical approach identified minimum predictive models, rather than mechanistic variables driving the process.

**Table S4.1.** A comparison of the global models for total cases and incidence. Total case data are reproduced from Table 3.

| **Scale** | **Incidence** | **Scaled RMSE** | ***R^2^*** | ***r_s_*** | ***r_p_*** |
| --- | --- | --- | --- | --- | --- |
| Subset | Y | 1.55 | 0.47 | 0.65 | 0.71 |
| Subset | N | 1.80 | 0.52 | 0.70 | 0.72 |
| All counties | Y | 3.49 | 0.11 | 0.40 | 0.34 |
| All counties | N | 2.45 | 0.72 | 0.39 | 0.86 |

**Table S4.2.** A list of the variables identified by the incidence models and the variables identified in the “Total Cases All counties” analysis. Total case variables for the surveillance subset are presented in Tables 4 and 5. Variable definitions are given in S2_File.

| Incidence Subset | Incidence All counties | Total Cases All counties |
| --- | --- | --- |
| IR | MEAN_MIN_T_3 | MEAN_MIN_T_3 |
| MAX_T_anomaly_2 | GDD10_anomaly_2 | MEAN_MAX_T_2 |
| MEAN_MAX_T_anomaly_2 | MEAN_MAX_T_anomaly_2 | MEAN_MIN_T_1 |
| TOTAL_POPULATION | Min_T_anomaly_2 |  |
|  | PercentUrban |  |
|  | TOTAL_POPULATION |  |
|  | MEAN_MAX_T_anomaly_1 |  |
