# Supplementary material for: Seasonal temperatures and hydrological conditions improve the prediction of West Nile virus infection rates in Culex mosquitoes and human case counts in New York and Connecticut
Source: PLoS One. 2019 Jun 3;14(6):e0217854. doi: 10.1371/journal.pone.0217854 (PMC6546252; doi:10.1371/journal.pone.0217854)
Supplement: S5 File — (DOCX) [file pone.0217854.s005.docx]

**Supplement S5 for:**

**Seasonal temperatures and hydrological conditions improve the prediction of West Nile virus infection rates in *Culex* mosquitoes and human case counts in New York and Connecticut**

Keyel, A.C., Elison Timm, O., Backenson, P.B., Prussing, C., Quinones, S., McDonough, K., Vuille, M., Conn, J.E., Armstrong, P.M., Andreadis, T.G., and Kramer, L.

**Sampling methods**

**Overall**

Across the entire data set, sampling began on average on June 8 and ended September 26, although starting dates varied by year and county (Table S5.1). The average number of pools (2 – 539) and average number of mosquitoes sampled (28 – 20957) varied substantially by county (Table S5.1).

**Table S5.1. Mean date of first and last sampling, number of pools, and number of mosquitoes for the season, averaged across each year the county was sampled.** Sampling dates are represented as day of year, with January 1 being day 1. In a non-leap year, 159 corresponds to June 8, and 269 corresponds to September 26.

| County | Mean first trap date | Mean last trap date | Mean number of pools | Mean number of mosquitoes |
| --- | --- | --- | --- | --- |
| ERIE, NY | 165 | 264 | 102 | 3455 |
| FAIRFIELD, CT | 154 | 287 | 539 | 8751 |
| HARTFORD, CT | 156 | 279 | 157 | 2441 |
| LITCHFIELD, CT | 160 | 248 | 19 | 86 |
| MIDDLESEX, CT | 157 | 268 | 52 | 382 |
| NASSAU, NY | 152 | 267 | 84 | 2450 |
| NEW HAVEN, CT | 154 | 284 | 272 | 3830 |
| NEW LONDON, CT | 156 | 283 | 154 | 2279 |
| ONEIDA, NY | 195 | 210 | 2 | 28 |
| ONONDAGA, NY | 179 | 234 | 13 | 336 |
| ORANGE, NY | 168 | 266 | 48 | 1350 |
| ROCKLAND, NY | 163 | 273 | 458 | 20957 |
| SUFFOLK, NY | 154 | 279 | 443 | 16015 |
| TOLLAND, CT | 161 | 265 | 34 | 328 |
| WESTCHESTER, NY | 152 | 267 | 159 | 3602 |
| WINDHAM, CT | 166 | 264 | 27 | 260 |
| ALL | 159 | 269 | 174 | 4389 |

**Connecticut**

The Connecticut sampling protocol has been previously described [1]. Briefly, the state of Connecticut maintains 91 permanent monitoring locations. Traps were typically set in the late afternoon and retrieved the following morning for each site. Each site was sampled at least every 10 days over the season. Live adult mosquitoes were transported to the Connecticut Agricultural Experimental Station laboratory, where they were identified to species using descriptions and keys [2–4]. Mosquitoes were then pooled by species, collection site, trap type, and date, with a maximum pool size of 50 and were tested for West Nile virus (see [1] for details).

Trap sites in Fairfield, New Haven, and Hartford counties were located in densely populated residential areas, in parks, greenways, golf courses, undeveloped wood lots, sewage treatment plants, dumping stations, and temporary wetlands associated with waterways. Trap sites in Litchfield, Middlesex, New London, Tolland, and Windham counties were in rural settings that included permanent fresh-water swamps and bogs, coastal salt marshes, horse stables, and swamp-forest border environs.

**New York**

Mosquito sampling methods varied by county in New York State. For all New York samples, mosquitoes are pooled by species, sex, date of collection, trap type, and location of collection into pools of up to 50 each, with a maximum of 90 pools per week for the duration of the transmission season. The pooling process is carried out individually by county, with only one county (Suffolk) submitting the maximum number of pools. Suffolk County preferentially submits *Culex pipens* and *Cx. restuans* for testing because of their interest in West Nile. Pools were submitted to the New York State Department of Health Arbovirus Laboratory (Wadsworth Center). Real-time reverse transcription-polymerase chain reaction (PCR) was used to identify WNV presence.

Mosquito collection methods from Suffolk county [5] have been previously published, while to our knowledge, the sampling protocols from Erie, Nassau, Oneida, Onondaga, Orange, Rockland, and Westchester have not been. In Suffolk County, gravid traps were baited with rabbit chow. Mosquito trapping was conducted from approximately early June to early October, but the exact timing depended on the mosquito populations and the presence of WNV. At the beginning of each season, trap sites were chosen based on previous presence of West Nile virus, and trap sites were expanded to include locations with positive humans, birds, or horses. Approximately half the traps were in or around town, county, or state parks. When mosquito numbers were low, mosquitoes from both gravid and light traps were combined, potentially over a period of two weeks. We excluded data from combined gravid and light traps.

Sampling protocols were not available for Erie, Nassau, Oneida, Onondaga, Rockland, Orange, and Westchester Counties. All traps in these locations were left out each night. As a consequence, baits were listed as unspecified. However, our analysis does not require specific assumptions about sampling effort, consequently the variations in sampling approach are not expected to invalidate the results obtained, but may contribute to the variation not explained by the model.

**Literature Cited**

1. Andreadis TG, Anderson JF, Vossbrinck CR, Main AJ. Epidemiology of West Nile virus in Connecticut: a five-year analysis of mosquito data 1999–2003. Vector-Borne Zoonotic Dis. 2004;4: 360–378.

2. Darsie R, Ward R. Identification and Geographical Distribution of the Mosquitoes of North America, North of Mexico. 1st ed. University Press of Florida, USA; 1981.

3. Means R. The Genus *Aedes* Meigen, with Identification Keys to Genera of Culicidae. N Y State Mus Bull. 1979;430a: 1–221.

4. Means R. Mosquitoes of New York: Part II. Genera of Culicidae other than *Aedes*. N Y State Mus Bull. 1987;430b: 1–180.

5. Shaman J, Harding K, Campbell SR. Meteorological and hydrological influences on the spatial and temporal prevalence of West Nile virus in Culex mosquitoes, Suffolk County, New York. J Med Entomol. 2011;48: 867–875.
