# Supplementary material for: Seasonal temperatures and hydrological conditions improve the prediction of West Nile virus infection rates in Culex mosquitoes and human case counts in New York and Connecticut
Source: PLoS One. 2019 Jun 3;14(6):e0217854. doi: 10.1371/journal.pone.0217854 (PMC6546252; doi:10.1371/journal.pone.0217854)
Supplement: S6 File — (DOCX) [file pone.0217854.s006.docx]

**Supplement S6 for:**

**Seasonal temperatures and hydrological conditions improve the prediction of West Nile virus infection rates in *Culex* mosquitoes and human case counts in New York and Connecticut**

Keyel, A.C., Elison Timm, O., Backenson, P.B., Prussing, C., Quinones, S., McDonough, K., Vuille, M., Conn, J.E., Armstrong, P.M., Andreadis, T.G., and Kramer, L.

**Comparison of climate data based on centroid and based on an average**

We used county centroids in our county-scale analysis. An alternative would have been to use the average of all grid cells within a county. Therefore, we compared the centroid results to an average of all grid centers that fell within a county boundary. The model results did not appreciably differ between the two analyses, with the climate data taken from the centroid possibly having more predictive value for West Nile than the climate data taken from an average of grid cells (Table S6.1).

**Table S6.1. A comparison of selected models for the centroid and the average approach for predicting human cases across all counties in NY and CT (882 county × year records).**

| **Method** | **RMSE** | **Median RMSE** | **Scaled RMSE** | **Max Error** | ***R^2^*** | ***r_s_*** | ***r_p_*** |
| --- | --- | --- | --- | --- | --- | --- | --- |
| Centroid | 2.1 | 1.57 | 2.45 | 30.2 | 0.72 | 0.39 | 0.86 |
| Average | 2.1 | 1.83 | 2.52 | 22.7 | 0.71 | 0.41 | 0.84 |

Both the Centroid and Average approaches identified Mean Minimum Temperature from July to September and Mean Maximum Temperature from April – June. The Centroid model identified Mean Minimum Temperature from January to March, while the Average model identified Minimum Temperature from January to March (i.e., the single lowest value from that time period rather than the average of all of the low values). Below we examine differences for Mean Minimum Temperature from July to September and Mean Maximum Temperature from April – June, as these variables were in common in both models and explained most of the variation in the data.

When the differences between data sets were averaged across all years for each county, most counties differed by less than 0.5 °C. Only 12% of counties for mean minimum temperature (Jul – Sep) and 18% of counties for mean maximum temperature (Apr – Jun) exceeded a mean difference of 0.5 °C (Fig. S6.1). Differences for some, if not all, counties are due to elevational differences (e.g., the Catskill mountains in Greene and the Adirondack mountains in Essex).

| 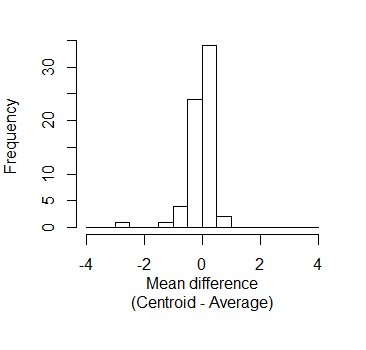  a. | 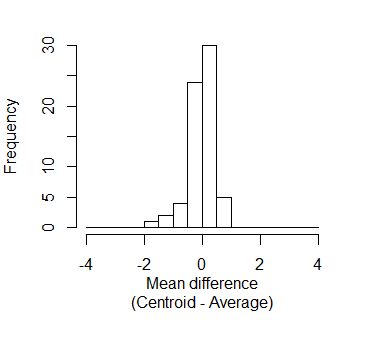  b. |
| --- | --- |
| 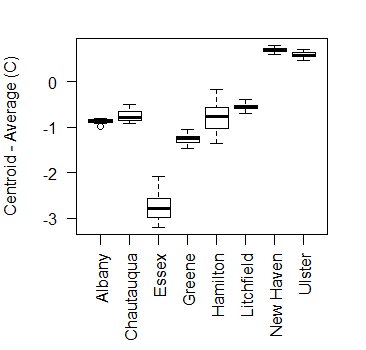  c. | 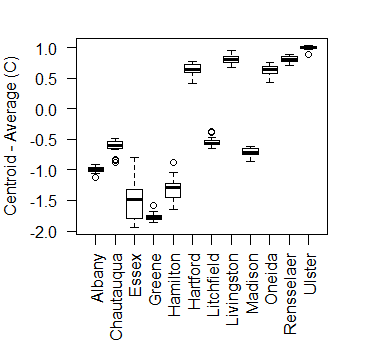  d. |

**Fig. S6.1.** The distribution of differences between the centroid and average data sets for **a.** Mean Minimum Temperature (Jul – Sep) and **b.** Mean Max Temperature (Apr – Jun). **c.** The deviations for the counties that differed by more than 0.5 °C between data sets for mean minimum temperature (Jul – Sep, 8 counties), and **d.** mean maximum temperature (Apr – Jun, 12 counties). Bar indicates the median, the box the 25% and 75% quartiles, the whiskers the min and max excluding any outliers. Outliers were plotted individually, and were defined as points that were more than 1.5 times the interquartile range from the 25% or 75% quartiles.
